# Supplementary material for: Breaking Bad News: A Simulation-Based Training Program for OB/GYN Residents
Source: MedEdPORTAL. 2026 Jun 4;22:11606. doi: 10.15766/mep_2374-8265.11606 (PMC13233813; doi:10.15766/mep_2374-8265.11606)
Supplement: Supplementary file 1 — Palliative Care Didactic.pptxCase 1 - Previable Preterm Prelabor Rupture.docxCase 2 - Surgical Complication.docxCase 3 - Cancer Diagnosis.docxCase 4 - Intrauterine Fetal Demise.docxPre- and Postsession Questionnaires.docx [file mep_2374-8265.11606-s001.zip › A. Palliative Care Didactic.pptx]

## Slide 1
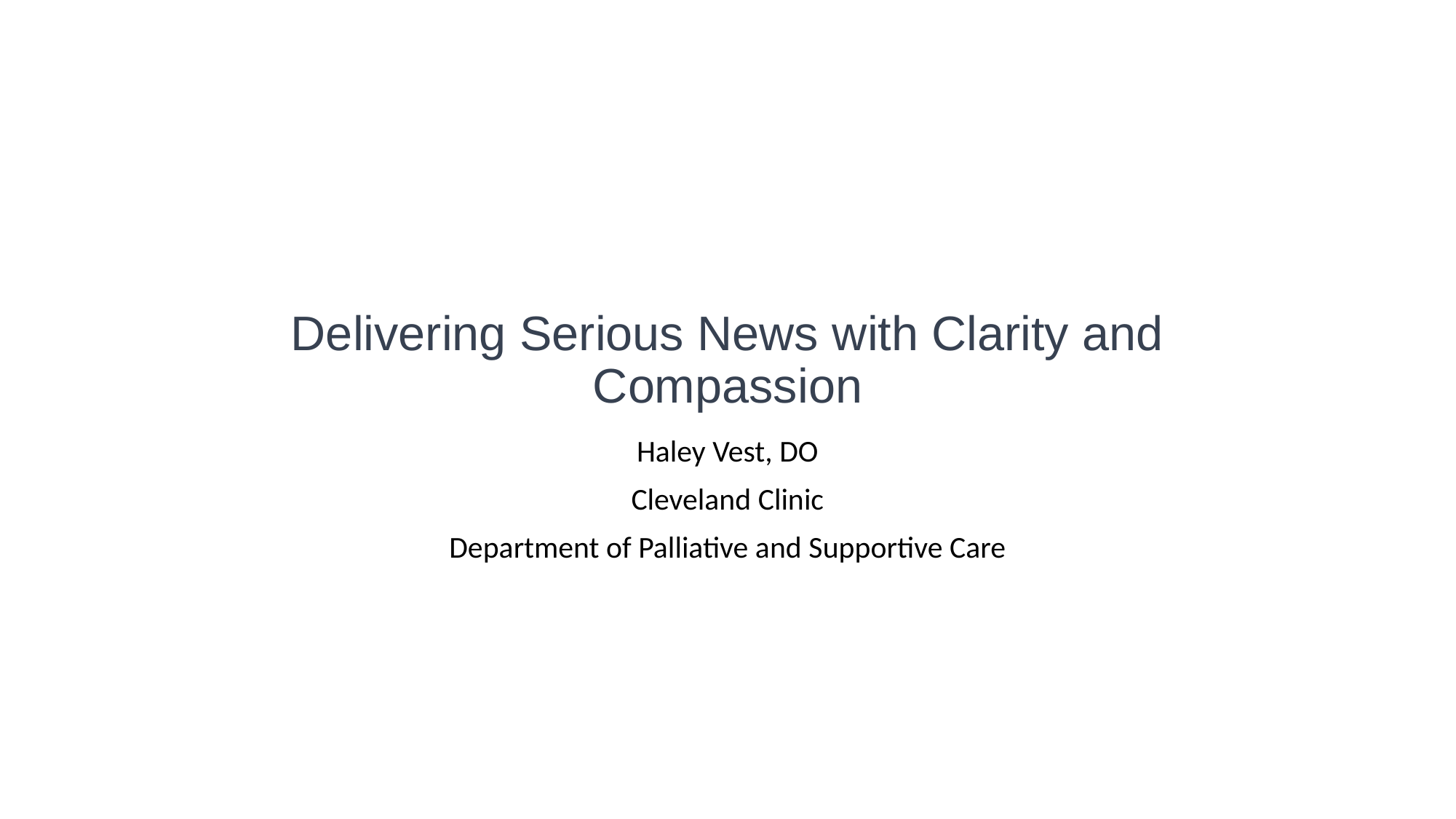

# Delivering Serious News with Clarity and Compassion
Haley Vest, DO
Cleveland Clinic
Department of Palliative and Supportive Care

## Slide 2
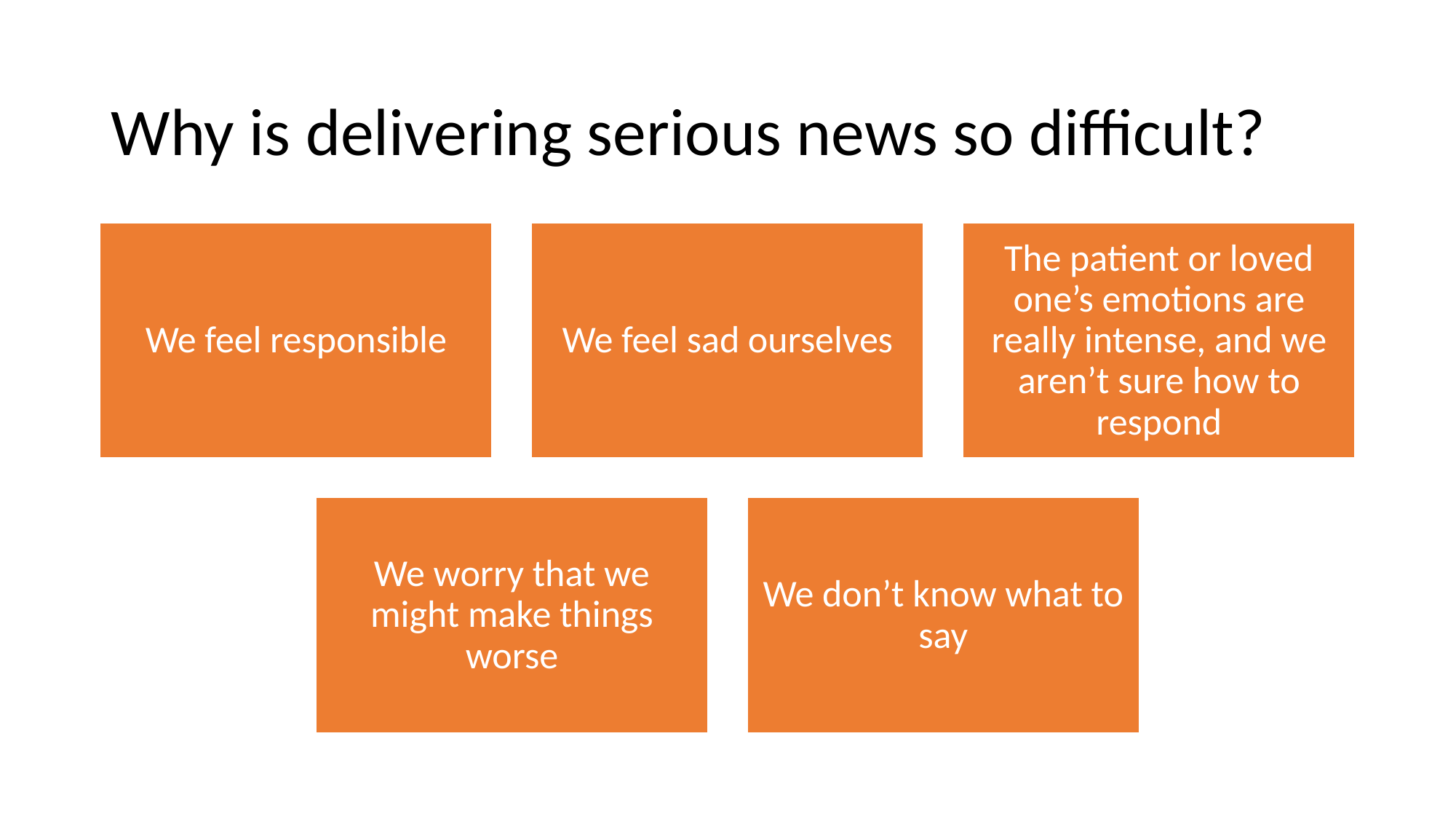

# Why is delivering serious news so difficult?
We feel responsible
We feel sad ourselves
The patient or loved one’s emotions are really intense, and we aren’t sure how to respond
We worry that we might make things worse
We don’t know what to say

## Slide 3
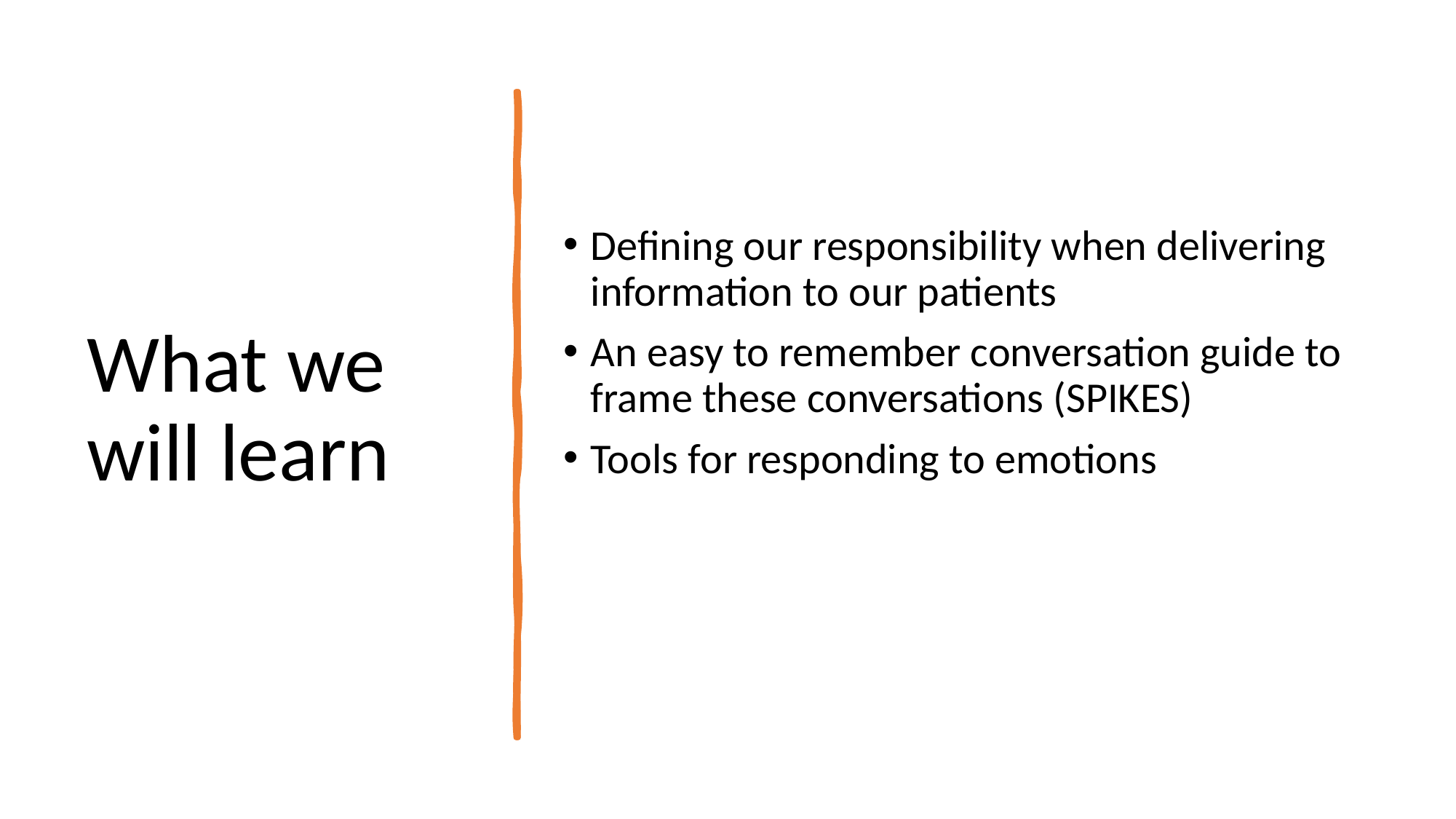

# What we will learn
Defining our responsibility when delivering information to our patients
An easy to remember conversation guide to frame these conversations (SPIKES)
Tools for responding to emotions

## Slide 4
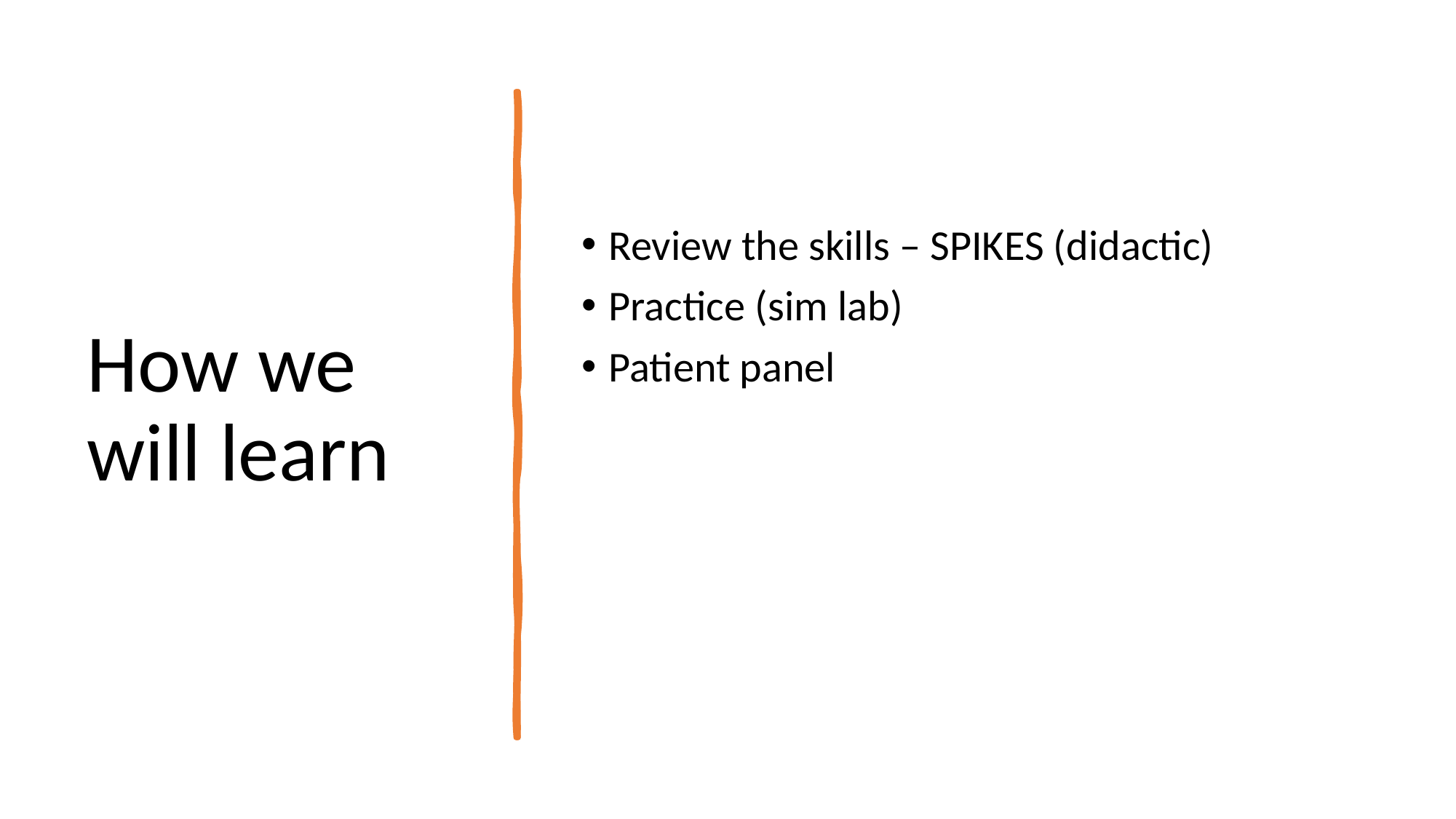

# How we will learn
Review the skills – SPIKES (didactic)
Practice (sim lab)
Patient panel

## Slide 5
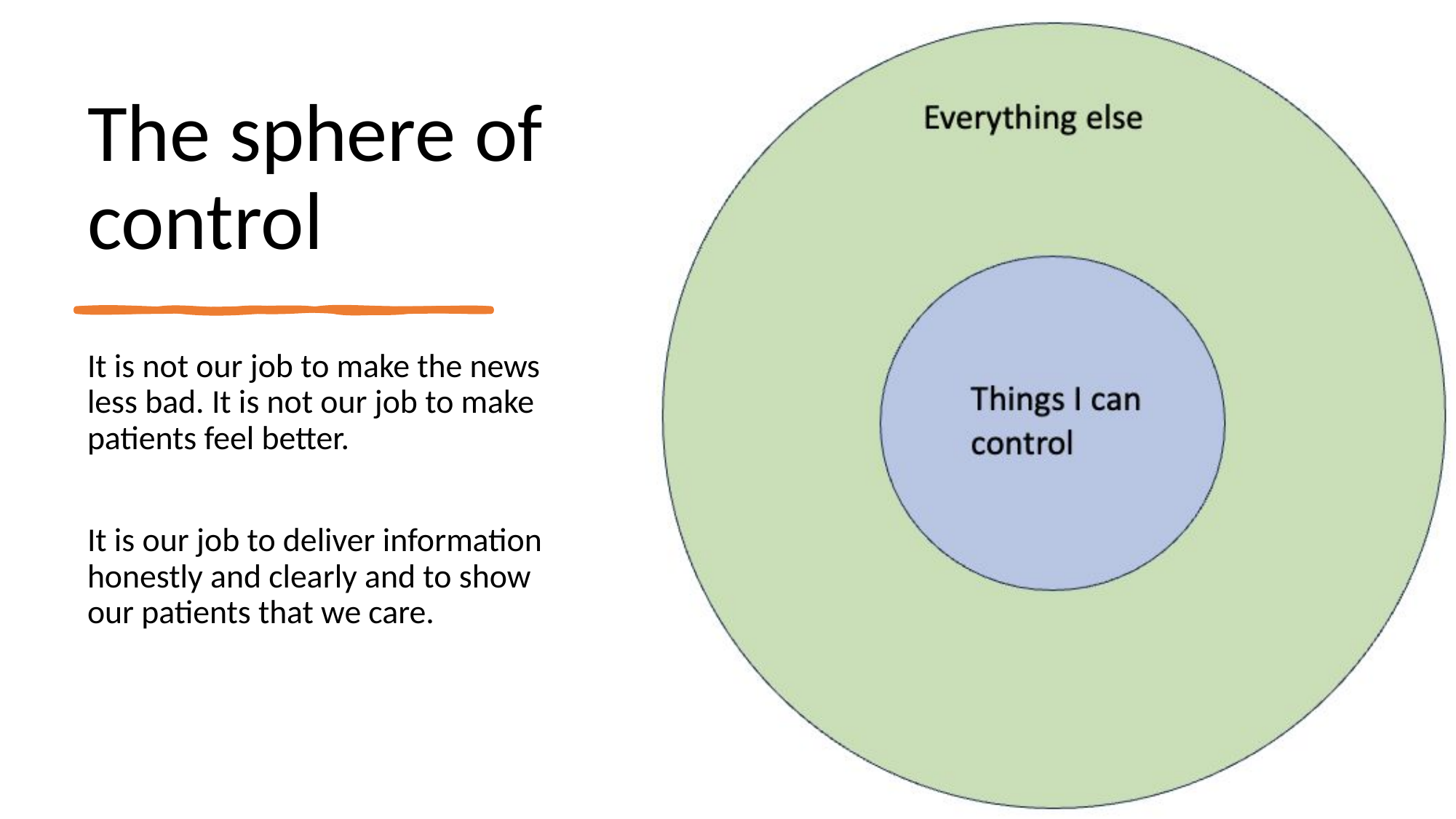

# The sphere of control
It is not our job to make the news less bad. It is not our job to make patients feel better.
It is our job to deliver information honestly and clearly and to show our patients that we care.

## Slide 6
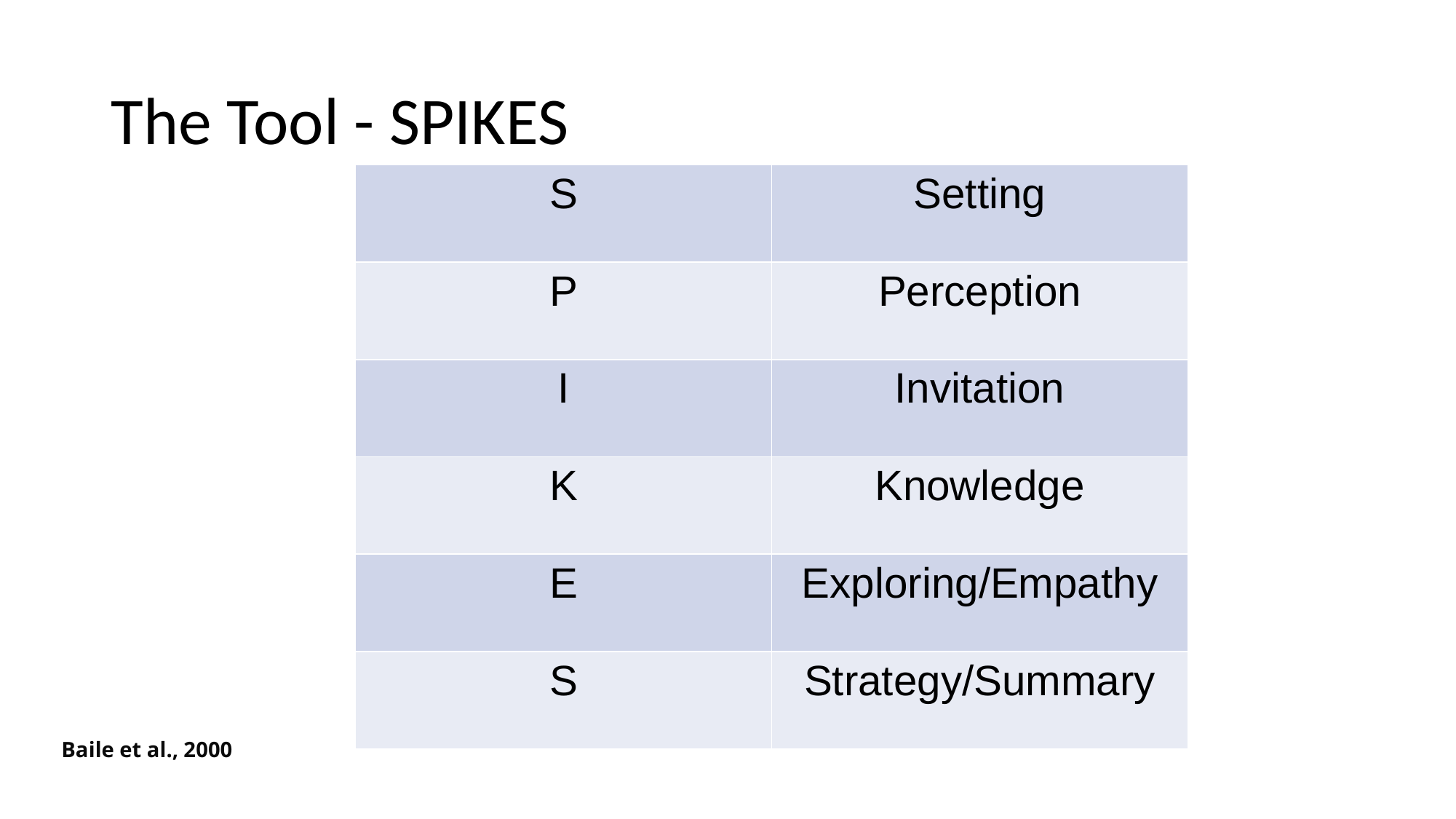

# The Tool - SPIKES
| S | Setting |
| --- | --- |
| P | Perception |
| I | Invitation |
| K | Knowledge |
| E | Exploring/Empathy |
| S | Strategy/Summary |
Baile et al., 2000

## Slide 7
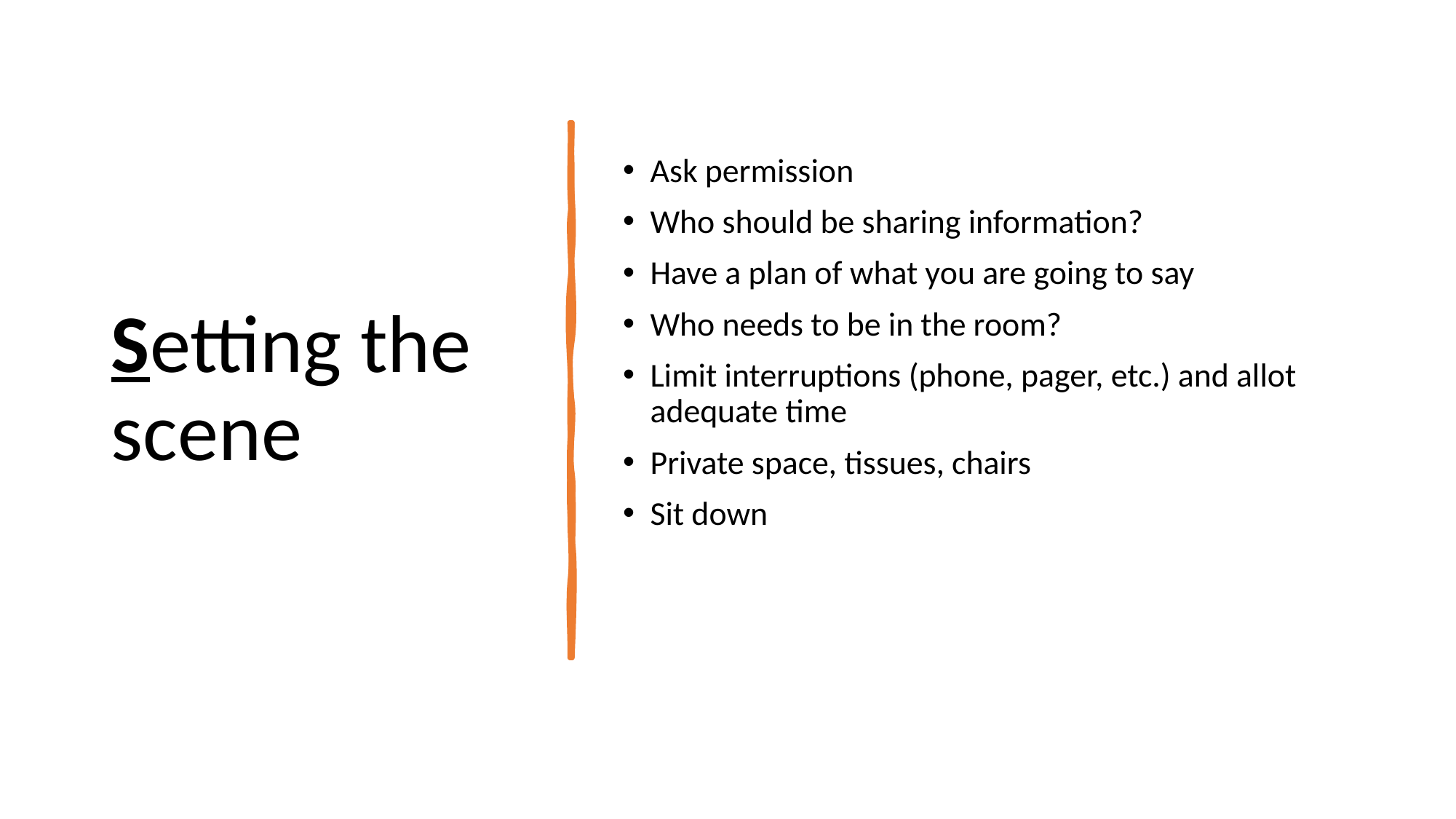

# Setting the scene
Ask permission
Who should be sharing information?
Have a plan of what you are going to say
Who needs to be in the room?
Limit interruptions (phone, pager, etc.) and allot adequate time
Private space, tissues, chairs
Sit down

## Slide 8
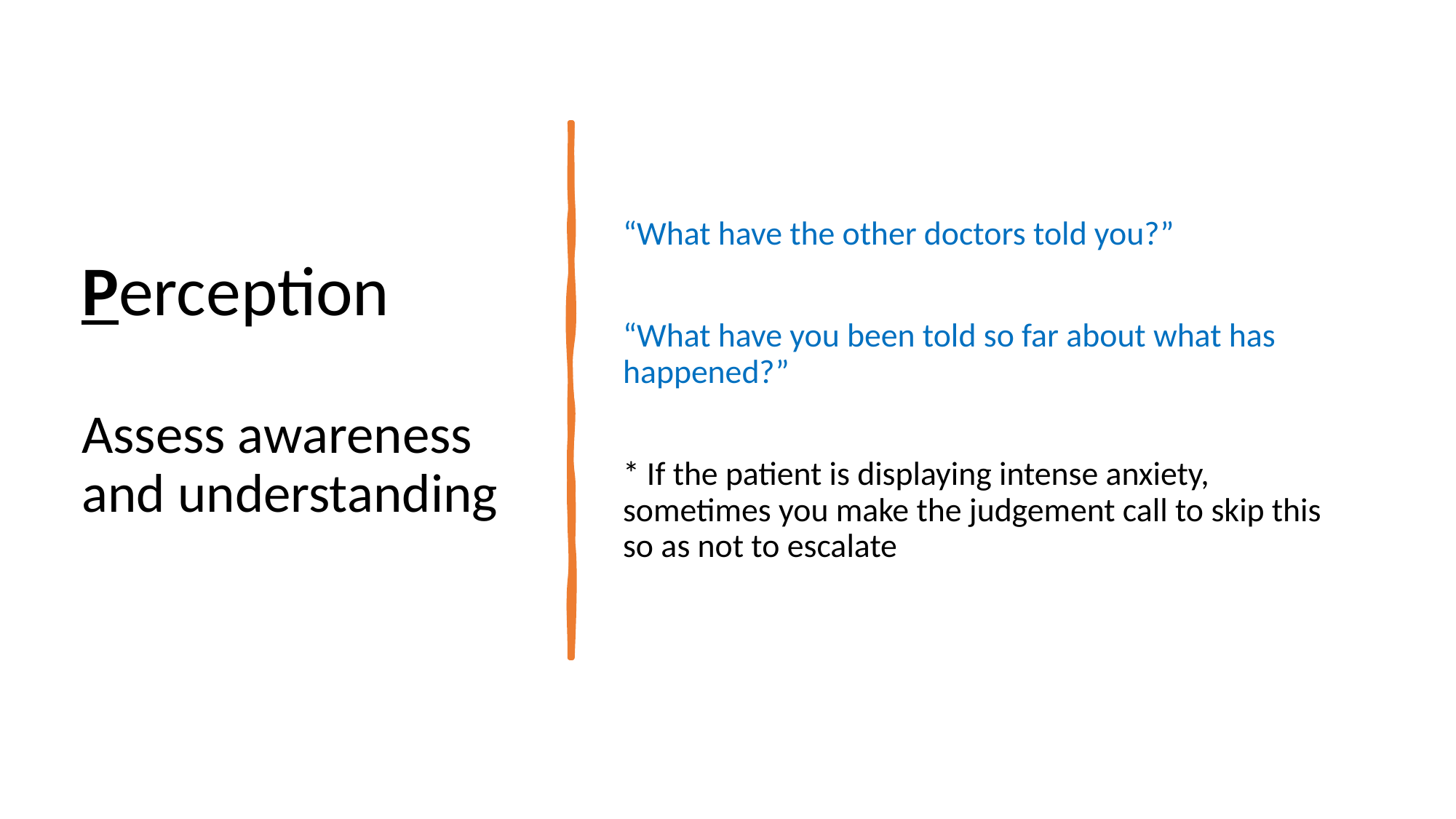

# Perception Assess awareness and understanding
“What have the other doctors told you?”
“What have you been told so far about what has happened?”
* If the patient is displaying intense anxiety, sometimes you make the judgement call to skip this so as not to escalate

## Slide 9
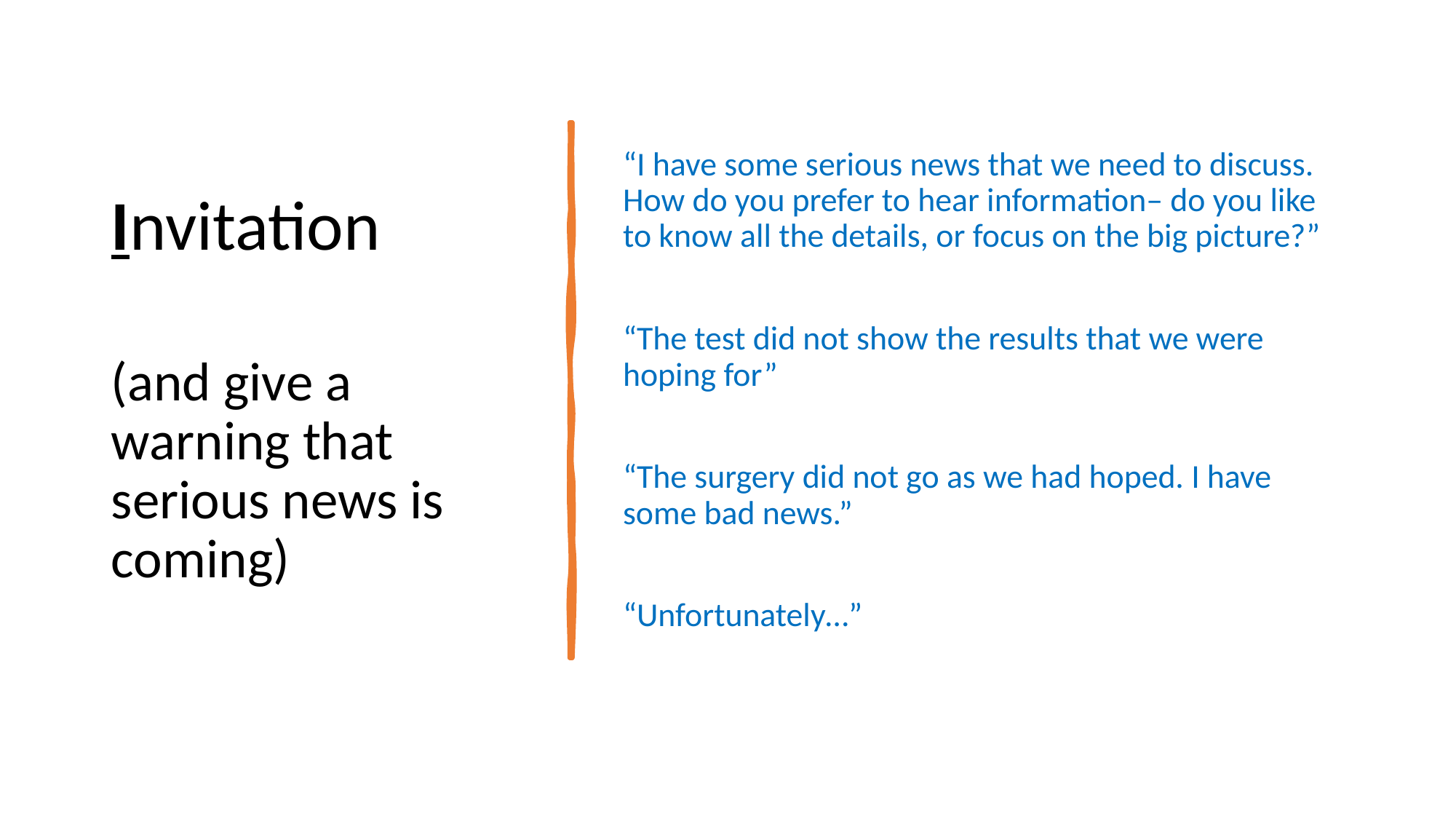

# Invitation (and give a warning that serious news is coming)
“I have some serious news that we need to discuss. How do you prefer to hear information– do you like to know all the details, or focus on the big picture?”
“The test did not show the results that we were hoping for”
“The surgery did not go as we had hoped. I have some bad news.”
“Unfortunately…”

## Slide 10
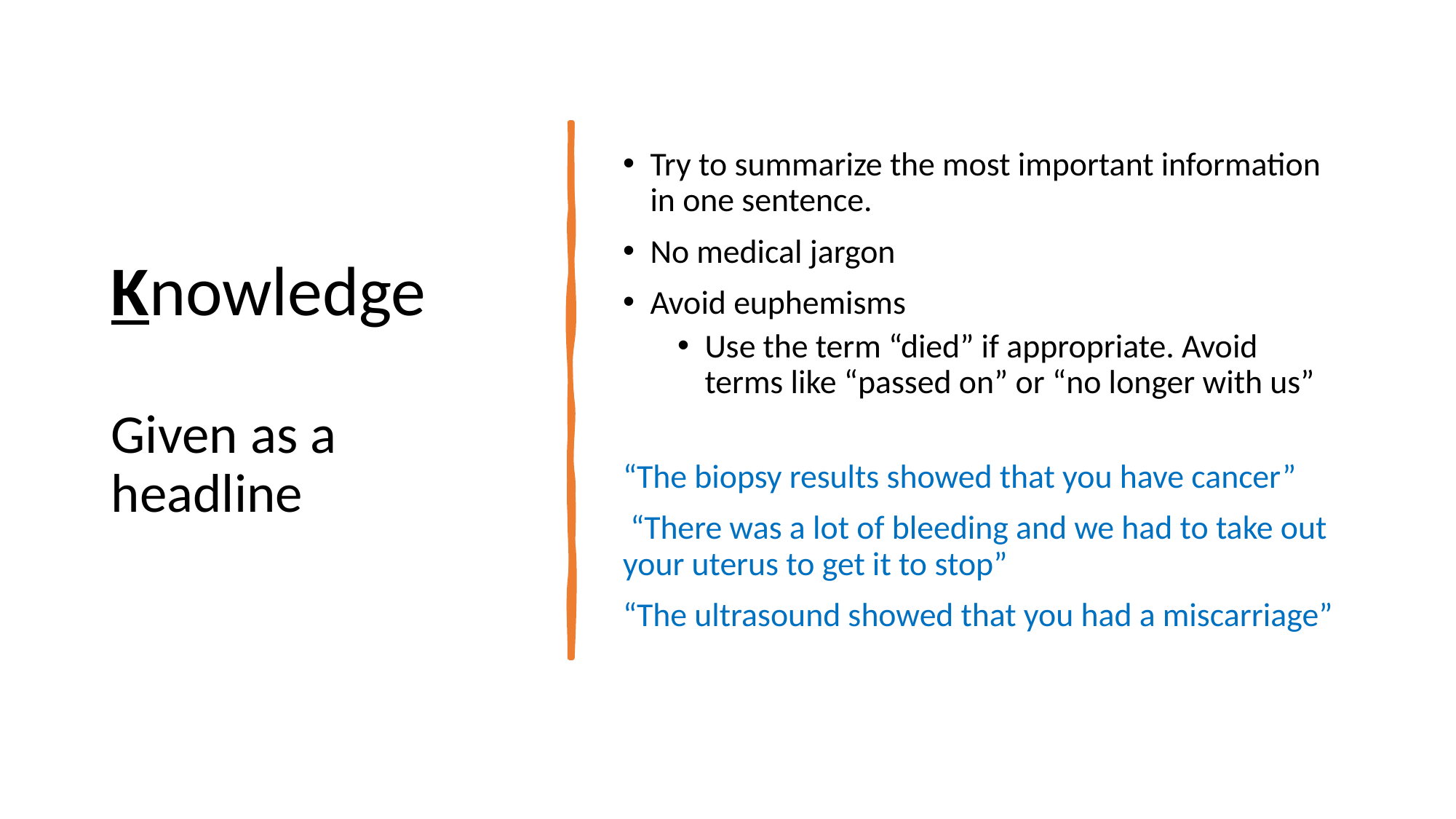

# Knowledge Given as a headline
Try to summarize the most important information in one sentence.
No medical jargon
Avoid euphemisms
Use the term “died” if appropriate. Avoid terms like “passed on” or “no longer with us”
“The biopsy results showed that you have cancer”
 “There was a lot of bleeding and we had to take out your uterus to get it to stop”
“The ultrasound showed that you had a miscarriage”

## Slide 11
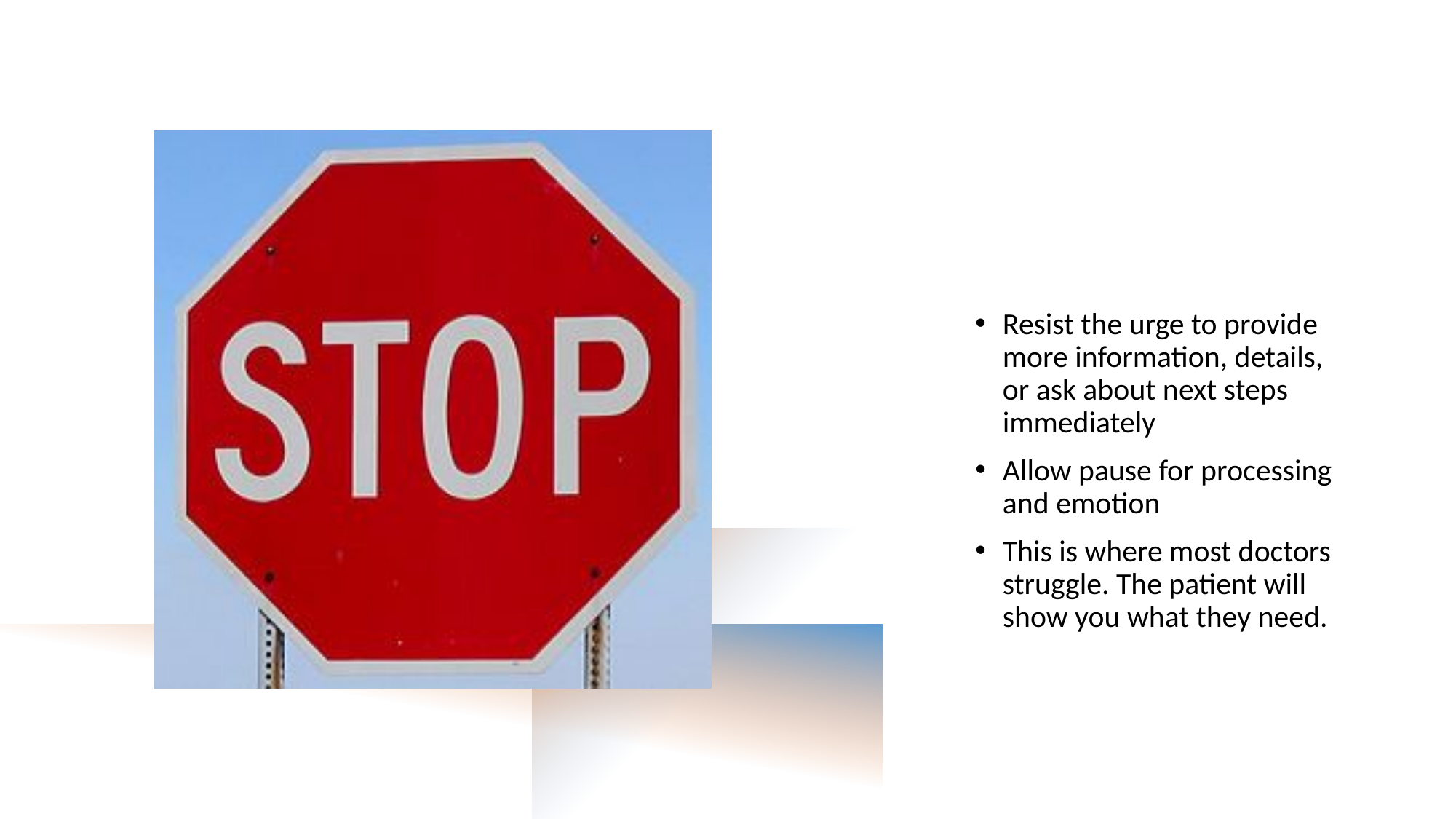

Resist the urge to provide more information, details, or ask about next steps immediately
Allow pause for processing and emotion
This is where most doctors struggle. The patient will show you what they need.

## Slide 12
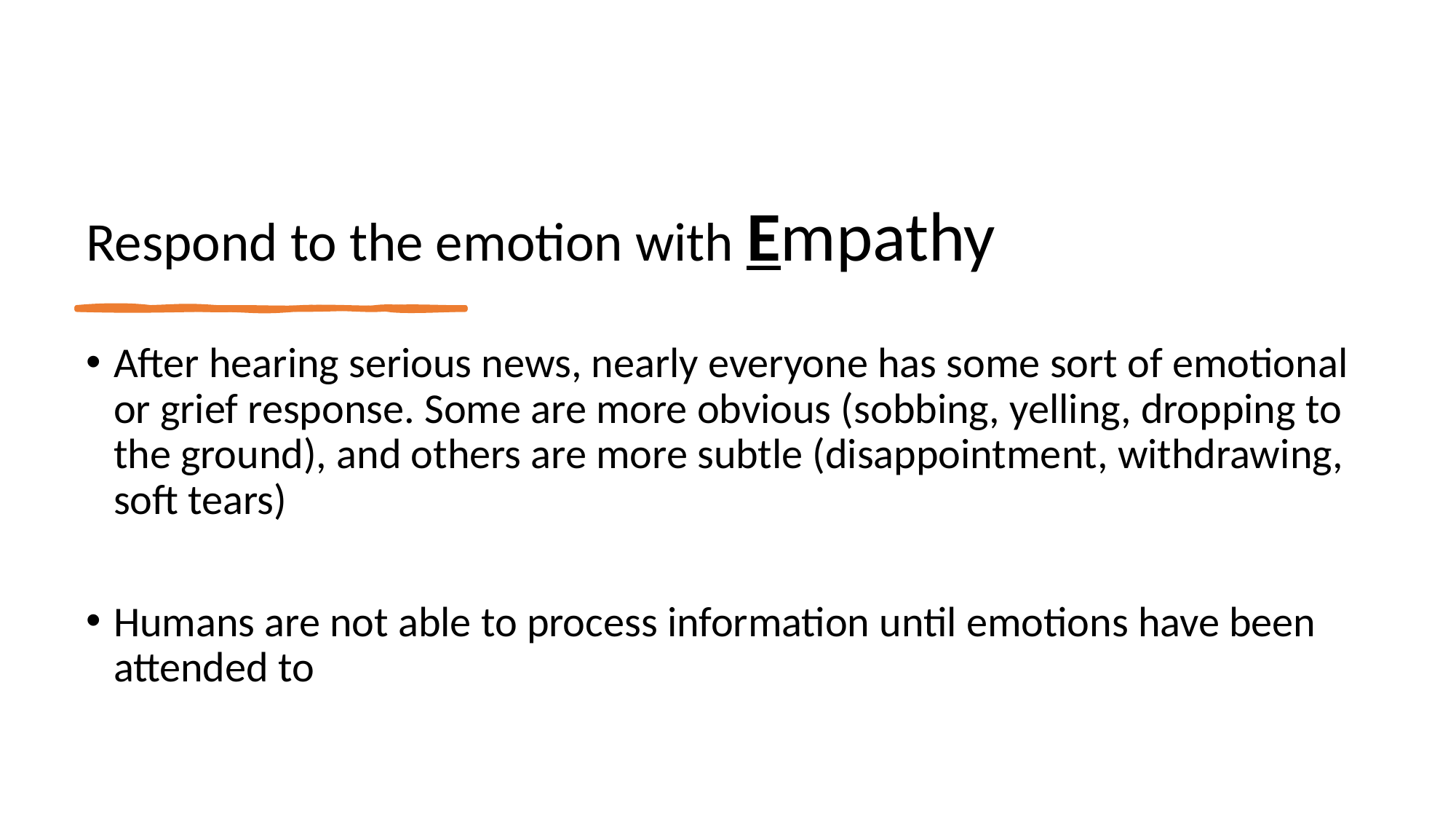

# Respond to the emotion with Empathy
After hearing serious news, nearly everyone has some sort of emotional or grief response. Some are more obvious (sobbing, yelling, dropping to the ground), and others are more subtle (disappointment, withdrawing, soft tears)
Humans are not able to process information until emotions have been attended to

## Slide 13
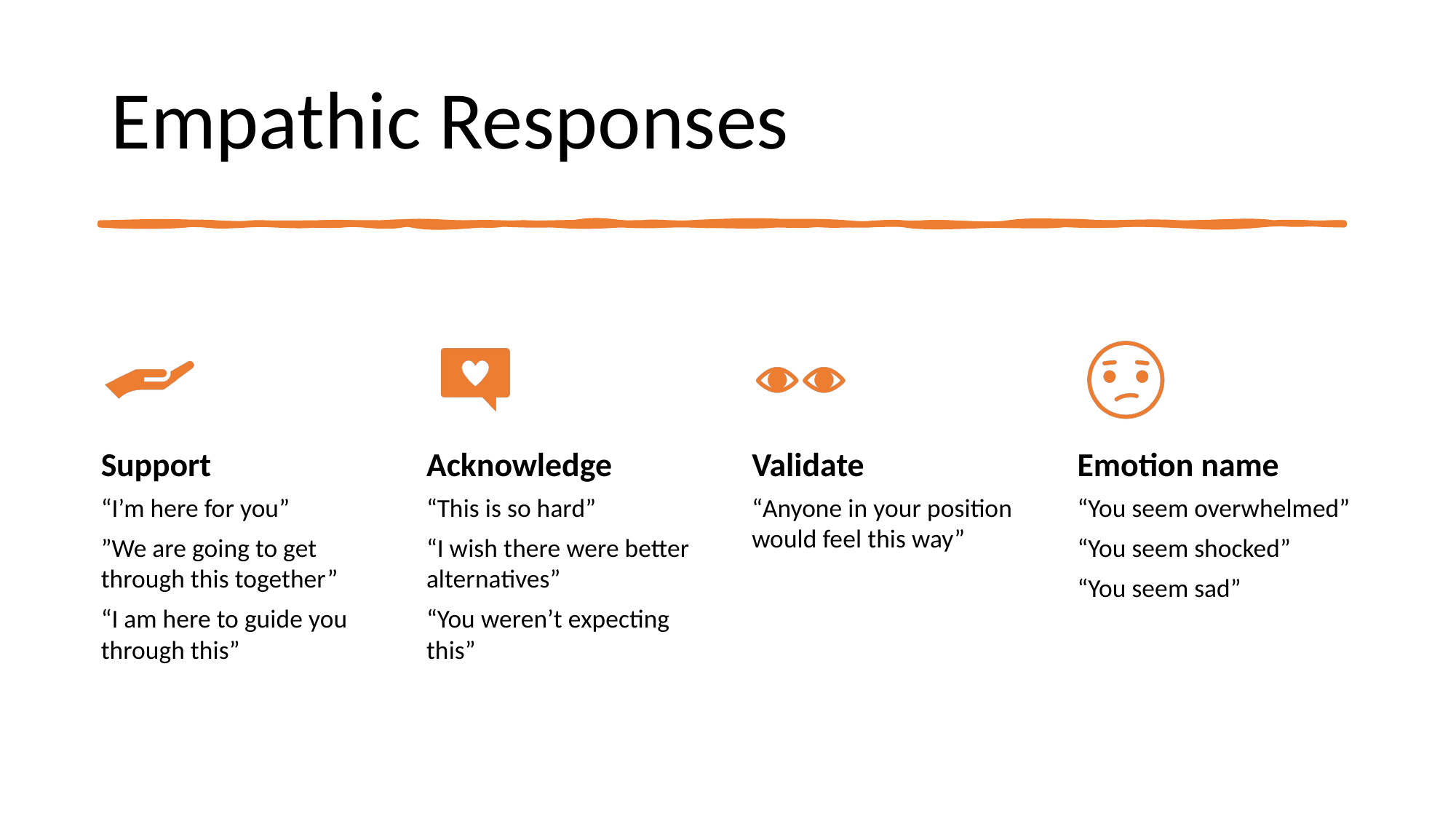

# Empathic Responses
Support
Acknowledge
Validate
Emotion name
“I’m here for you”
”We are going to get through this together”
“I am here to guide you through this”
“This is so hard”
“I wish there were better alternatives”
“You weren’t expecting this”
“Anyone in your position would feel this way”
“You seem overwhelmed”
“You seem shocked”
“You seem sad”

## Slide 14
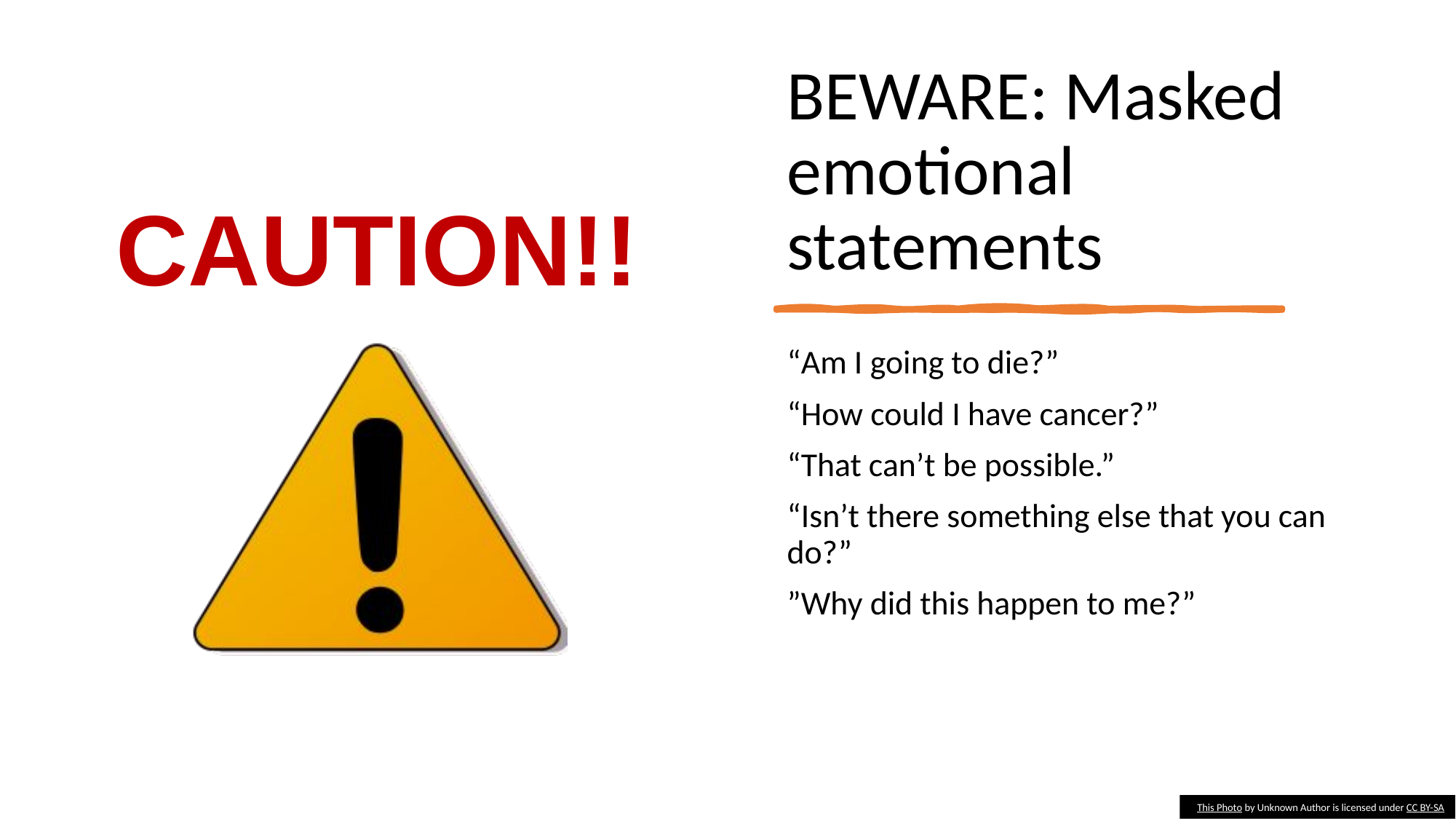

# BEWARE: Masked emotional statements
CAUTION!!
“Am I going to die?”
“How could I have cancer?”
“That can’t be possible.”
“Isn’t there something else that you can do?”
”Why did this happen to me?”
This Photo by Unknown Author is licensed under CC BY-SA

## Slide 15
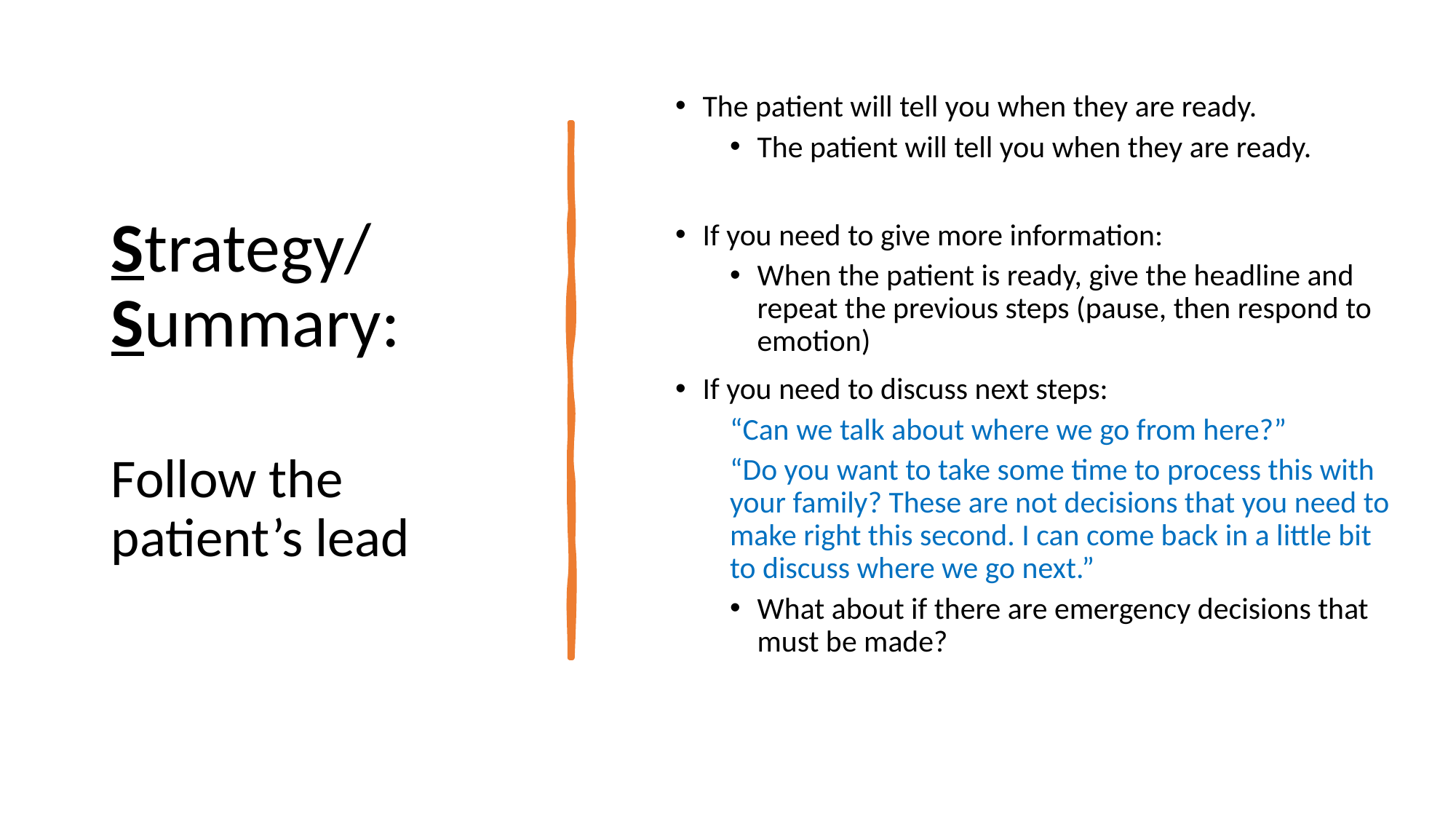

# Strategy/ Summary: Follow the patient’s lead
The patient will tell you when they are ready.
The patient will tell you when they are ready.
If you need to give more information:
When the patient is ready, give the headline and repeat the previous steps (pause, then respond to emotion)
If you need to discuss next steps:
“Can we talk about where we go from here?”
“Do you want to take some time to process this with your family? These are not decisions that you need to make right this second. I can come back in a little bit to discuss where we go next.”
What about if there are emergency decisions that must be made?

## Slide 16
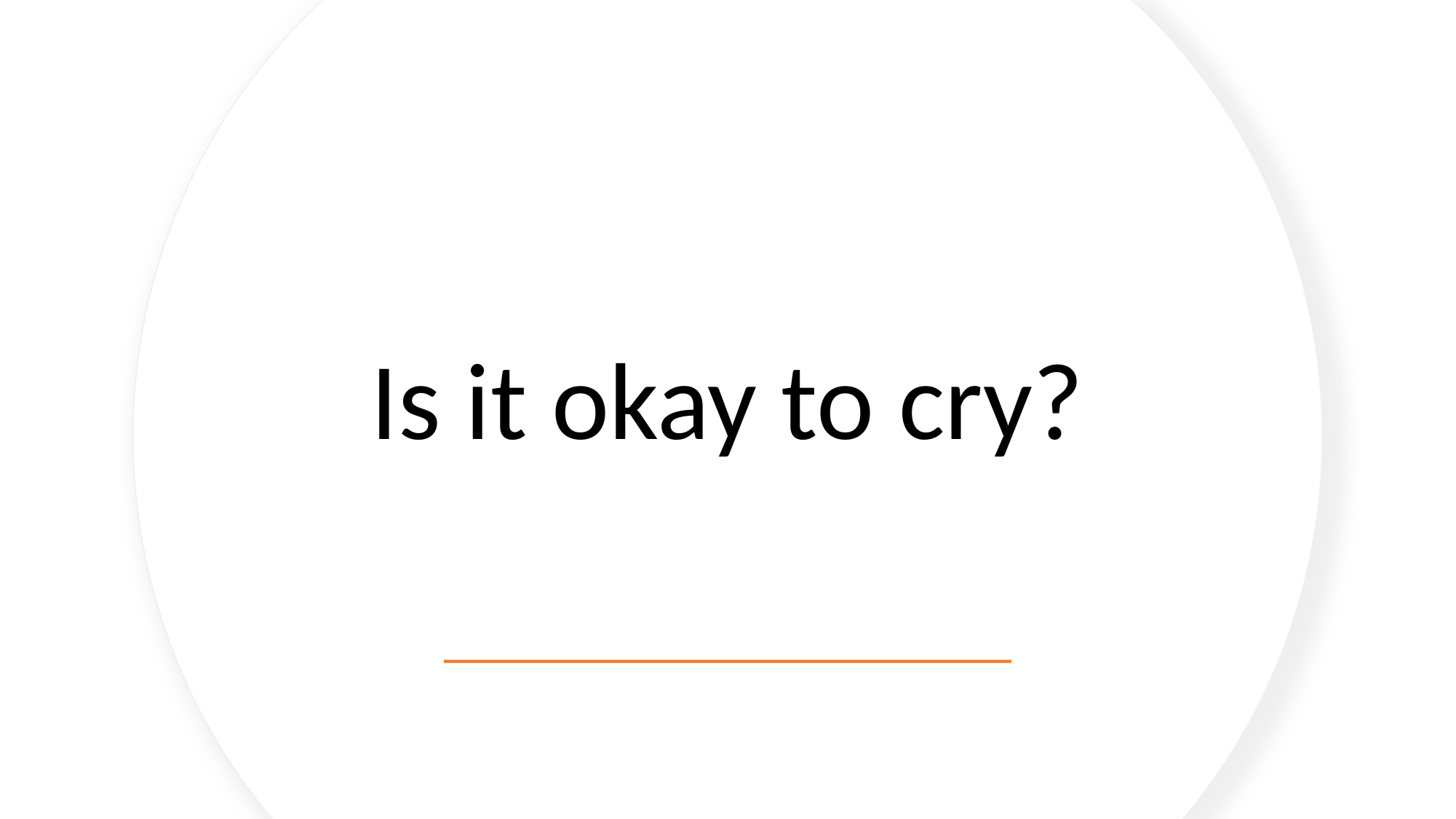

# Is it okay to cry?

## Slide 17
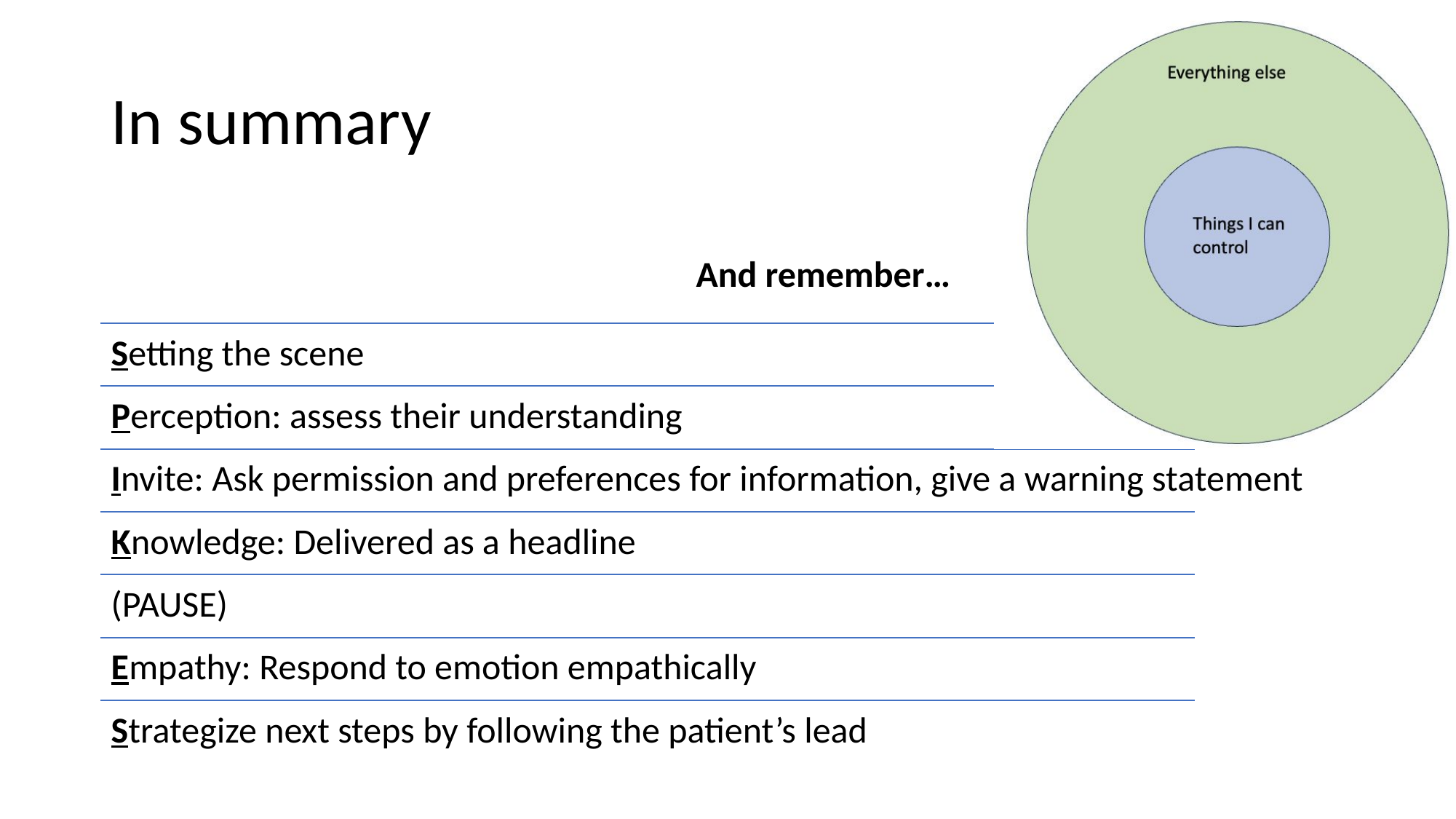

# In summary
And remember…
Setting the scene
Perception: assess their understanding
Invite: Ask permission and preferences for information, give a warning statement
Knowledge: Delivered as a headline
(PAUSE)
Empathy: Respond to emotion empathically
Strategize next steps by following the patient’s lead

## Slide 18
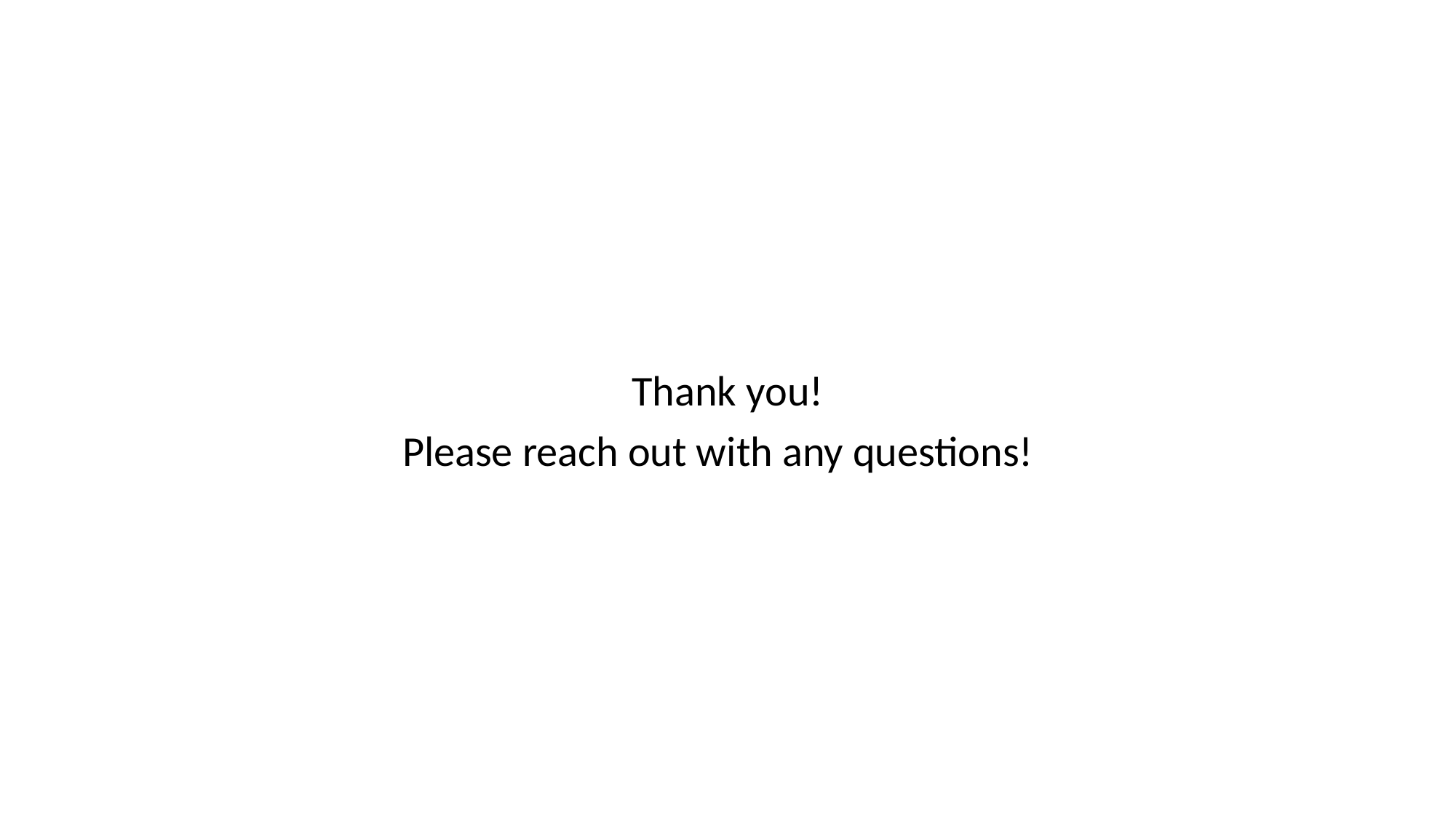

Thank you!
Please reach out with any questions!
