# Supplementary material for: Breaking Bad News: A Simulation-Based Training Program for OB/GYN Residents
Source: MedEdPORTAL. 2026 Jun 4;22:11606. doi: 10.15766/mep_2374-8265.11606 (PMC13233813; doi:10.15766/mep_2374-8265.11606)
Supplement: Supplementary file 1 — Palliative Care Didactic.pptxCase 1 - Previable Preterm Prelabor Rupture.docxCase 2 - Surgical Complication.docxCase 3 - Cancer Diagnosis.docxCase 4 - Intrauterine Fetal Demise.docxPre- and Postsession Questionnaires.docx [file mep_2374-8265.11606-s001.zip › B. Case 1 - Previable Preterm Prelabor Rupture.docx]

Date: January 2025

Primary Case Author: Jonathan Seibert, MD

Secondary Case Author: Erin Higgins, MD

Standardized Patient Educator: Sarah Barton, MBA

Name of Case: Previable PPROM

Name of Educational Activity: Breaking Bad News: A Simulation-based Training Program for Ob/Gyn Residents

Patient Name: Ellie Evans

Chief Complaint: “I think my water broke”

Most Likely Diagnosis and Differential With Rationale From History and/or Physical Exam:

1. PPROM- Most likely diagnosis given clinical history (“pop and large gush of fluid” while patient on toilet), exam (*not conducted during encounter)* with obvious pooling of fluid in vaginal canal, confirmed positive for amniotic fluid.
2. Urinary incontinence- Less likely given large volume of fluid lost and exam/lab findings consistent with amniotic fluid.
3. Bacterial vaginosis- Less likely due to large volume of fluid lost at one time with sudden onset and lack of other symptoms (vaginal pruritus, odor).

Challenge Question: None

Domains: Check all that apply

□ Professionalism

X Communication and Interpersonal Skills

□ Medical History

□ Physical Exam

X Shared Decision-Making

X Patient Education

□ Clinical Reasoning

□ Documentation

□ Handoff

□ Presentation

□ Other:

Type and Level of Learner: Ob/Gyn Resident

Case Objectives:

1. Discuss the diagnosis of previable PPROM and its implications.
2. Counsel the patient on their options & associated risks (D&E vs. induction vs. expectant management).
3. Engage in shared decision-making with the patient to decide on a management option.
4. Address patient emotional concerns appropriately.

| SETTING | OB triage |
| --- | --- |
| PATIENT PROFILE | |
| Age range | 30 |
| Religious/spiritual background | She is religious and goes to church regularly |
| Sex | Female |
| Sexual orientation | Heterosexual |
| Gender expression | Woman |
| Race and ethnicity | All may be used |
| Physical description (e.g., BMI, height range) | All may be used |
| Physical limitations | All may be used |
| Patient appearance | In hospital gown |
| Moulage + location | Gravid abdomen consistent with 19 weeks gestation |
| Affect | She is a bubbly extroverted person |
| Family group | She lives at home with her husband and two daughters |
| Education | BSN |
| Level of health literacy | RN who has been in pediatrics practice for several years |
| Employment | She is a nurse at a pediatrics office, has been working in same office since graduating with her BSN |
| Home | She lives at home with her husband and two daughters, any type of home may be used |
| Financial situation | All may be used |
| Insurance status | Private |
| Habits | All may be used for diet/exercise, but patient has never used tobacco, alcohol, or other drugs |
| Activities | All may be used; patient religious and regularly attends church |
| Typical day | Going to work as pediatric RN, taking care of her two daughters, attending church on Sunday |

| CASE INFORMATION | |
| --- | --- |
| Chief Concern | “I think my water broke” |
| Additional Concerns | None |
| THE PATIENT’S STORY | ***Do not disclose personal information unless asked directly***  The patient is in Ob triage. She is wearing a hospital gown. The Ob intern has performed a speculum exam that confirms PPROM.  The patient is a 30 year old female who is 19 weeks pregnant. She has had two prior pregnancies that were uncomplicated for which she had two vaginal deliveries. She has no medical problems. She is excited because she has two girls and this is going to be her first boy.  She was sitting on the toilet this morning when she heard a pop and felt a gush of fluid. She thought maybe it was just urine and didn’t think much of it. After breakfast she noticed her underwear were completely wet. She changed her underwear, put a pad on, and came straight to the OB triage to get evaluated.  She has no abdominal pain. She is feeling her baby boy move. She hasn’t noticed any vaginal bleeding.  She is sitting on the hospital bed with a gown on. She has already been examined and is awaiting the results of her pelvic exam to see if her water is broken.  The resident comes in to discuss the results of her exam.  The patient will say: “Hopefully I just peed myself, right?”  **SP instructions:** Once the resident delivers the diagnosis of previable preterm premature rupture of membranes (PPROM), the patient will be confused and get progressively more tearful once she starts to understand what this diagnosis means. She will originally be against terminating/IOL as she is very religious, but after counseling, she will start to consider it.  She will ask:  “What does this mean?”  “Is my baby ok? Is my baby going to be ok?”  “What caused this? Why did this happen?”  “What did I do wrong?”  “What happens next?”  “Are you sure I can’t stay pregnant?”  “Are you sure this isn’t a mistake?” |
| HISTORY OF PRESENT ILLNESS | |
| Onset | This morning, sudden |
| Setting | Sitting on the toilet this morning when she heard a pop and felt a gush of fluid |
| Duration | After breakfast she noticed her underwear were completely wet. She changed her underwear, put a pad on, and came straight to the OB triage to get evaluated. |
| Time relationships |  |
| Location |  |
| Radiation |  |
| Quality |  |
| Amount | Fluid soaked underwear by end of breakfast |
| Aggravated by what |  |
| Relieved by what |  |
| Associated with what |  |
| Attitude | The patient is hoping that the fluid is urine but is very concerned that her water broke. |
| Overall course |  |
| REVIEW OF SYSTEMS | |
| +Fetal movement | - Abdominal pain |
|  | - Vaginal bleeding |
| Past medical history | |
| Medication allergies (name and reaction) | None |
| Environmental allergies (name and reaction) | None |
| Illnesses | None |
| Vaccinations | Up to date on vaccinations |
| Surgeries | None |
| Accidents/injuries/trauma | None |
| Hospitalization | None |
|  | |
| Inclusive sexual and reproductive history | |
| Sexual practices  Sexual partners  Protection: Use of safer sex practices  Use of birth control if appropriate  Risk of intimate partner violence | Monogamous with husband  1 sexual partner  No birth control or barrier contraception  No concerns for IPV |
| OB/GYN history | Age of onset of menses 14  Age of menopause NA  Number of pregnancies 3  Number of live births 2  Number of miscarriages 0  Number of abortions 0 |
| Medications | Prescription/dose/reason   - Prenatal vitamins, daily   Over the counter/dose/reason- NA  Herbs/supplements/dose/reason- NA |
| Immunizations | X Tetanus  X Flu  X Hepatitis  □ Pneumovax  X HPV  X Covid  □ Other |
| Tobacco products  □ Cigarettes  □ Cigar  □ Pipe  □ Chew  □ E-cigarettes | X Never  □ Past - year started/year quit  □ Current  o ppd  o # of years |
| Alcohol  □ Beer  □ Wine  □ Liquor  □ Other | X Never  □ Past - year started/year quit  □ Current  o Quantity  o # of years |
| Drugs  □ Weed  □ Cocaine  □ Heroin  □ Meth  □ IV  □ Inhalants  □ Other | X Never  □ Past - year started/year quit  □ Current  o Quantity  o # of years |
| Diet | All may be used |
| Exercise | All may be used |
| List any other important social history or information important to this case | NA |
| Family history | |
| Mother, father, siblings, grandparents, and others | NA |
|  |  |
| Physical Exam:  No physical exam conducted during this encounter; however findings communicated by resident to patient regarding physical exam are an exam consistent with previable PPROM (eg pooling of fluid in vaginal canal, +ferning and pH tests). | |
|  |  |
| DIAGNOSIS AND DIFFERENTIAL | |
| Diagnosis with support from positive and negative history and PE findings | PPROM- Most likely diagnosis given clinical history (“pop and large gush of fluid” while patient on toilet), exam (*not conducted during encounter)* with obvious pooling of fluid in vaginal canal, confirmed positive for amniotic fluid. |
| Differential with support from positive and negative history and PE findings | Urinary incontinence- Less likely given large volume of fluid lost and exam/lab findings consistent with amniotic fluid.  Bacterial vaginosis- Less likely due to large volume of fluid lost at one time with sudden onset and lack of other symptoms (vaginal pruritus, odor). |
|  |  |
| MANAGEMENT OR DIAGNOSTIC PLAN | |
|  | Discuss diagnosis of previable PPROM. Inform the patient that this is likely not a viable pregnancy and discuss D&E vs induction vs expectant management. Engage the patient in shared decision-making to agree on a management plan. |
| PROFESSIONALISM ISSUES OR CHALLENGES | Along with the challenge of needing to discuss that the pregnancy is not viable, the learner will need to navigate the patient’s religious beliefs in their counseling. Thus, they will need to be professional in medical recommendations while balancing the patient’s concerns regarding terminating the pregnancy or inducing labor, especially given that the fetus is still alive. |
