# Supplementary material for: Breaking Bad News: A Simulation-Based Training Program for OB/GYN Residents
Source: MedEdPORTAL. 2026 Jun 4;22:11606. doi: 10.15766/mep_2374-8265.11606 (PMC13233813; doi:10.15766/mep_2374-8265.11606)
Supplement: Supplementary file 1 — Palliative Care Didactic.pptxCase 1 - Previable Preterm Prelabor Rupture.docxCase 2 - Surgical Complication.docxCase 3 - Cancer Diagnosis.docxCase 4 - Intrauterine Fetal Demise.docxPre- and Postsession Questionnaires.docx [file mep_2374-8265.11606-s001.zip › C. Case 2 - Surgical Complication.docx]

Date: January 2025

Primary Case Author: Jonathan Seibert, MD

Secondary Case Author: Erin Higgins, MD

Standardized Patient Educator: Sarah Barton, MBA

Name of Case: Surgical Complication

Name of Educational and/or Assessment Activity: Breaking Bad News: A Simulation-based Training Program for Ob/Gyn Residents

Patient Name: Theresa Kho

Chief Complaint: Post-op following scheduled total laparoscopic hysterectomy and bilateral salpingectomy for surgical management of adenomyosis.

Most Likely Diagnosis and Differential With Rationale From History and/or Physical Exam:

1. Adenomyosis- Most likely diagnosis given patient with known history of this condition for which she is undergoing surgical management; previously seen on imaging.
2. Endometrial hyperplasia- Less likely given patient’s premenopausal status with consistent heavy, painful menstruation and imaging consistent with adenomyosis.
3. Endometrial cancer-Less likely given patient’s premenopausal status with consistent heavy, painful menstruation and imaging consistent with adenomyosis.

Challenge Question: None

Domains: Check all that apply

□ Professionalism

X Communication and Interpersonal Skills

□ Medical History

□ Physical Exam

□ Shared Decision-Making

X Patient Education

□ Clinical Reasoning

□ Documentation

□ Handoff

□ Presentation

□ Other:

Type and Level of Learner: Ob/Gyn resident

Case Objectives:

1. Disclose the surgical complication to the patient and her partner with the appropriate level of sincerity and empathy.
2. Educate the patient on the surgical complication, steps taken in the operating room, and any future implications.
3. Address the patient and partner’s emotions, reactions, and questions.
4. Discuss the plan moving forward with the patient.

| SETTING | PACU |
| --- | --- |
| PATIENT PROFILE | |
| Age range | 50 |
| Religious/spiritual background | All may be used |
| Sex | Female |
| Sexual orientation | Lesbian or bisexual |
| Gender expression | Woman |
| Race and ethnicity | All may be used |
| Physical description | All may be used |
| Physical limitations | All may be used |
| Patient appearance | Hospital gown, uncomfortable and in pain following surgery |
| Moulage + location | Large bandage on abdomen |
| Affect | Very matter of fact; likes people to be straightforward with her |
| Family group | She lives at home with her partner Barb and their two cats |
| Education | All may be used |
| Level of health literacy | All may be used |
| Employment | All may be used |
| Home | She lives at home with her partner Barb and their two cats; any home type can be used |
| Financial situation | All may be used |
| Insurance status | All may be used |
| Habits | She drinks socially, does not smoke or use drugs. She regularly exercises. |
| Activities | She is a very active person and loves to play tennis, bike, and swim |
| Typical day | All may be used, usually involves some sort of exercise |

| CASE INFORMATION | |
| --- | --- |
| Chief Concern | Post-op following scheduled total laparoscopic hysterectomy and bilateral salpingectomy for surgical management of adenomyosis. |
| Additional Concerns | None |
| THE PATIENT’S STORY | ***Do not disclose personal information unless asked directly***  The patient is in the PACU about 90 minutes post-op. She is joined by her partner Barb who was her “support person” for her surgery. She is alert and awake, wearing a hospital gown. She has one large bandage on her abdomen.  The patient is a 50 year old female who has a history of abnormal uterine bleeding secondary to adenomyosis and who strongly desired surgical management.  She underwent her scheduled surgery earlier today and is recovering in the PACU. During abdominal entry, there was a bowel injury requiring conversion to an open case (instead of a laparoscopic surgery). The patient is not aware of this yet.  The plan was originally for her to go home today but the nurse in the PACU is telling her she has to stay overnight and she’s confused and very frustrated. She is very uncomfortable with a foley in her bladder and wants to take it out but her nurse won’t let her which is making her more frustrated. She also sees a big incision on her abdomen and she thought she was going to just have small incisions on her abdomen.  The resident comes into the room to discuss the surgery (and unanticipated complication) with the patient.  “The nurse is saying that I’m not allowed to go home and that I can’t take this foley out of my goddamn bladder. Can you please tell her she’s wrong?”  **SP instructions:** Once the resident discloses the bowel injury and complication, the patient will become visibly upset and angry. She will state that she never would have gotten the surgery if she had known something like this could happen and mentions that she is going to hire a lawyer.  She will state things along the lines of  “How could this happen to a qualified surgeon? Explain to me exactly how this mistake could happen”  “Why did this happen?”  “I never wanted to stay in the hospital and now you’re telling me I’m going to be here for several days!?”  “What’s my recovery going to look like now? I can’t take any time off of work!”  The patient's partner, Barb, will also be present.  Barb will act upset that the complication happened. She cares about the patient and is very protective of her and would never want her to get hurt. To Barb, this mistake feels like their partner was hurt, and she doesn’t really care that it was unintentional because it still happened. She might get angry or frustrated and continue to ask why this happened and what can be done to fix it. Barb will let the patient ask most of the questions but still make it clear that she is upset. At the same time, she will act in a loving and comforting way to the patient. |
| HISTORY OF PRESENT ILLNESS | |
| Onset |  |
| Setting |  |
| Duration |  |
| Time relationships |  |
| Location |  |
| Radiation |  |
| Quality |  |
| Amount |  |
| Aggravated by what |  |
| Relieved by what |  |
| Associated with what |  |
| Attitude |  |
| Overall course |  |
| REVIEW OF SYSTEMS | |
| +Abdominal Pain |  |
| +Abdominal Bloating |  |
| +Nausea/vomiting |  |
| Past medical history | |
| Medication allergies (name and reaction) | None |
| Environmental allergies (name and reaction) | None |
| Illnesses | HTN, ulcerative colitis, anemia |
| Vaccinations | Up to date on vaccinations |
| Surgeries | Ulcerative colitis with h/o bowel resection |
| Accidents/injuries/trauma | None |
| Hospitalization | None |
|  | |
| Inclusive sexual and reproductive history | |
| Sexual practices  Sexual partners  Protection: Use of safer sex practices  Use of birth control if appropriate  Risk of intimate partner violence | Sexually active with 1 female partner, Barb  No contraception or barrier protection  No concerns of IPV |
| OB/GYN history | Age of onset of menses 15  Age of menopause Perimenopausal  Number of pregnancies 0  Number of live births 0  Number of miscarriages 0  Number of abortions 0 |
| Medications | Prescription/dose/reason   - Ulcerative colitis: tofacitinib - HTN: lisinopril - Anemia: PO ferrous sulfate   Over the counter/dose/reason- NA  Herbs/supplements/dose/reason- NA |
| Immunizations | X Tetanus  X Flu  X Hepatitis  X Pneumovax  X COVID  □ HPV  □ Other |
| Tobacco products  □ Cigarettes  □ Cigar  □ Pipe  □ Chew  □ E-cigarettes | X Never  □ Past - year started/year quit  □ Current  o ppd  o # of years |
| Alcohol  □ Beer  X Wine  □ Liquor  □ Other | □ Never  □ Past - year started/year quit  X Current  o Socially |
| Drugs  □ Weed  □ Cocaine  □ Heroin  □ Meth  □ IV  □ Inhalants  □ Other | X Never  □ Past - year started/year quit  □ Current  o Quantity  o # of years |
| Diet | All may be used |
| Exercise | She is a very active person and loves to play tennis, bike, and swim |
| List any other important social history or information important to this case | NA |
| Family history |  |
| Mother, father, siblings, grandparents, and other significant findings | NA |
|  |  |
| Physical Exam  No physical exam conducted | |
|  |  |
| DIAGNOSIS AND DIFFERENTIAL | |
| Diagnosis with support from positive and negative history and PE findings | Adenomyosis- Most likely diagnosis given patient with known history of this condition for which she is undergoing surgical management; previously seen on imaging. |
| Differential with support from positive and negative history and PE findings | Endometrial hyperplasia- Less likely given patient’s premenopausal status with consistent heavy, painful menstruation and imaging consistent with adenomyosis.  Endometrial cancer-Less likely given patient’s premenopausal status with consistent heavy, painful menstruation and imaging consistent with adenomyosis. |
|  |  |
| MANAGEMENT OR DIAGNOSTIC PLAN | |
|  | Disclose the surgical complication with an appropriate level of sincerity and empathy. Discuss the plan moving forward.  The patient will understand the complication and eventually become less upset with more counseling. |
| PROFESSIONALISM ISSUES OR CHALLENGES | The challenge faced by learners with this SP encounter is being able to deliver objective information regarding a surgical complication while balancing patient emotions. Additional challenges are the patient discussing taking legal action against the surgery team and sharing information with the patient’s partner who is also in the room. |
