# Supplementary material for: Breaking Bad News: A Simulation-Based Training Program for OB/GYN Residents
Source: MedEdPORTAL. 2026 Jun 4;22:11606. doi: 10.15766/mep_2374-8265.11606 (PMC13233813; doi:10.15766/mep_2374-8265.11606)
Supplement: Supplementary file 1 — Palliative Care Didactic.pptxCase 1 - Previable Preterm Prelabor Rupture.docxCase 2 - Surgical Complication.docxCase 3 - Cancer Diagnosis.docxCase 4 - Intrauterine Fetal Demise.docxPre- and Postsession Questionnaires.docx [file mep_2374-8265.11606-s001.zip › D. Case 3 - Cancer Diagnosis.docx]

Date: January 2025

Primary Case Author: Jonathan Seibert, MD

Secondary Case Author: Erin Higgins, MD

Standardized Patient Educator: Sarah Barton, MBA

Name of Case: Cancer Diagnosis

Name of Educational and/or Assessment Activity: Breaking Bad News: A Simulation-based Training Program for Ob/Gyn Residents

Patient Name: Patty Stark

Chief Complaint: Abdominal pain, abdominal bloating, nausea and vomiting x 3 months

Most Likely Diagnosis and Differential With Rationale From History and/or Physical Exam:

1. Ovarian Cancer: Most likely diagnosis given ovarian mass on CT A/P, elevated CA-125 level, and symptoms (abdominal pain, abdominal bloating, and nausea/vomiting).
2. Diverticulosis: Less likely diagnosis given imaging and lab findings, in differential given symptoms of abdominal pain and bloating, chronic in nature.
3. Gastritis: Less likely diagnosis given imaging and lab findings as well as symptom duration of 3 months, and more likely to present with acute-onset nausea/vomiting and abdominal pain.

Challenge Question: None

Domains: Check all that apply

□ Professionalism

X Communication and Interpersonal Skills

□ Medical History

□ Physical Exam

□ Shared Decision-Making

X Patient Education

□ Clinical Reasoning

□ Documentation

□ Handoff

□ Presentation

□ Other:

Type and Level of Learner: Ob/Gyn resident

Case Objectives:

1. Discuss a metastatic cancer diagnosis with a patient, educating the patient on the implications of the diagnosis.
2. Address the patient’s emotional needs when given the diagnosis rather than focusing on the medical management.
3. Gain comfort in addressing patient questions when you may not have all of the answers.

| SETTING | Outpatient office |
| --- | --- |
| PATIENT PROFILE | |
| Age range | 65 |
| Religious/spiritual background | All may be used |
| Sex | Female |
| Sexual orientation | All may be used |
| Gender expression | Woman |
| Race and ethnicity | All may be used |
| Physical description | All may be used |
| Physical limitations | All may be used |
| Patient appearance | Street clothes |
| Moulage + location | None |
| Affect | Composed and quiet but very anxious |
| Family group | She lives at home alone. Her husband passed away several years ago. She has three children and 9 grandchildren. |
| Education | All may be used |
| Level of health literacy | All may be used |
| Employment | She is a retired accountant |
| Home | She lives at home alone; any home type can be used |
| Financial situation | All may be used |
| Insurance status | Medicare |
| Habits | All may be used |
| Activities | All may be used |
| Typical day | All may be used |

| CASE INFORMATION | |
| --- | --- |
| Chief Concern | Abdominal pain, abdominal bloating, nausea and vomiting x 3 months |
| Additional Concerns | None |
| THE PATIENT’S STORY | ***Do not disclose personal information unless asked directly***  The patient is at the office to follow up after an ED visit for abdominal pain, bloating, and nausea/vomiting that she’s been having for 3 months. She underwent a CT abdomen / pelvis at the ED which demonstrated a large 10cm ovarian mass suspicious for malignancy along with presumed metastasis to her liver and lungs. Her CA-125 was elevated. She underwent a paracentesis for ascites in the ED and that fluid was sent to be tested for malignant cells to confirm a cancer diagnosis. She is here at the office to find out the results of the paracentesis.  The patient’s first statement is: “I’m so worried about this diagnosis, I have barely been able to sleep.”  **SP instructions:** Once the resident discloses the diagnosis of metastatic ovarian cancer, the patient will be tearful and appropriately upset.  When resident delivers diagnosis:  “What does this mean?”  “Am I dying?”  “How long do I have to live?”  When resident describes spread of diagnosis:  “Does this mean that I have cancer all over my body?”  “What are the treatment options?”  “When can we start treatment?”  When resident asks what questions the patient has:  “Is there something I could have done to prevent this?”  “Why is this happening to me?” |
| HISTORY OF PRESENT ILLNESS | |
| Onset |  |
| Setting |  |
| Duration | Abdominal pain, abdominal bloating, nausea and vomiting x 3 months |
| Time relationships |  |
| Location |  |
| Radiation |  |
| Quality |  |
| Amount |  |
| Aggravated by what |  |
| Relieved by what |  |
| Associated with what |  |
| Attitude |  |
| Overall course |  |
| REVIEW OF SYSTEMS | |
| + Abdominal Pain |  |
| + Abdominal Bloating |  |
| + Nausea/vomiting |  |
| Past medical history | |
| Medication allergies (name and reaction) | None |
| Environmental allergies (name and reaction) | None |
| Illnesses | HTN, HLD, GERD |
| Vaccinations | Up to date on vaccinations |
| Surgeries | H/o cesarean section x 3  H/o cholecystectomy |
| Accidents/injuries/trauma | None |
| Hospitalization | None |
|  | |
| Inclusive sexual and reproductive history | |
| Sexual practices  Sexual partners  Protection: Use of safer sex practices  Use of birth control if appropriate  Risk of intimate partner violence | Not sexually active  No risk of IPV |
| OB/GYN history | Age of onset of menses 14  Age of menopause 51  Number of pregnancies 3  Number of live births 3  Number of miscarriages 0  Number of abortions 0 |
| Medications | Prescription/dose/reason   - HTN: lisinopril - HLD: atorvastatin - GERD: pantoprazole   Over the counter/dose/reason- NA  Herbs/supplements/dose/reason- NA |
| Immunizations | X Tetanus  X Flu  X Hepatitis  X Pneumovax  X COVID  □ HPV  □ Other |
| Tobacco products  □ Cigarettes  □ Cigar  □ Pipe  □ Chew  □ E-cigarettes | X Never  □ Past - year started/year quit  □ Current  o ppd  o # of years |
| Alcohol  □ Beer  □ Wine  □ Liquor  □ Other | X Never  □ Past - year started/year quit  □ Current  o Quantity  o # of years |
| Drugs  □ Weed  □ Cocaine  □ Heroin  □ Meth  □ IV  □ Inhalants  □ Other | X Never  □ Past - year started/year quit  □ Current  o Quantity  o # of years |
| Diet | All may be used |
| Exercise | All may be used |
| List any other important social history or information important to this case | NA |
| Family history | |
| Mother, father, siblings, grandparents, and other significant findings | Mother: h/o breast cancer at age 50  Father: HTN  MGM: h/o ovarian cancer at age 51 |
|  |  |
| Physical Exam  No physical exam conducted | |
|  |  |
| DIAGNOSIS AND DIFFERENTIAL | |
| Diagnosis with support from positive and negative history and PE findings | Ovarian Cancer: Most likely diagnosis given ovarian mass on CT A/P, elevated CA-125 level, and symptoms (abdominal pain, abdominal bloating, and nausea/vomiting). |
| Differential with support from positive and negative history and PE findings | Diverticulosis: Less likely diagnosis given imaging and lab findings, in differential given symptoms of abdominal pain and bloating, chronic in nature.  Gastritis: Less likely diagnosis given imaging and lab findings as well as symptom duration of 3 months, and more likely to present with acute-onset nausea/vomiting and abdominal pain. |
|  |  |
| MANAGEMENT OR DIAGNOSTIC PLAN | |
|  | Discuss diagnosis of metastatic ovarian cancer. Address emotional concerns first. If time allows, you can discuss treatment options, but this is not a necessity. |
| PROFESSIONALISM ISSUES OR CHALLENGES | The main challenge faced by learners in this case is discussing a terminal diagnosis with a patient. Learners must be able to navigate addressing the patient’s emotional concerns while being honest about the diagnosis. |
