# Supplementary material for: Breaking Bad News: A Simulation-Based Training Program for OB/GYN Residents
Source: MedEdPORTAL. 2026 Jun 4;22:11606. doi: 10.15766/mep_2374-8265.11606 (PMC13233813; doi:10.15766/mep_2374-8265.11606)
Supplement: Supplementary file 1 — Palliative Care Didactic.pptxCase 1 - Previable Preterm Prelabor Rupture.docxCase 2 - Surgical Complication.docxCase 3 - Cancer Diagnosis.docxCase 4 - Intrauterine Fetal Demise.docxPre- and Postsession Questionnaires.docx [file mep_2374-8265.11606-s001.zip › E. Case 4 - Intrauterine Fetal Demise.docx]

Date: January 2025

Primary Case Author: Jonathan Seibert, MD

Secondary Case Author: Erin Higgins, MD

Standardized Patient Educator: Sarah Barton, MBA

Name of Case: IUFD

Name of Educational and/or Assessment Activity: Breaking Bad News: A Simulation-based Training Program for Ob/Gyn Residents

Patient Name: Rebecca Smith

Chief Complaint: Decreased fetal movement

Most Likely Diagnosis and Differential With Rationale From History and/or Physical Exam:

1. Intrauterine fetal demise: Most likely diagnosis given clinical history of decreased fetal movement and lack of a heartbeat detected on Doppler and ultrasound.
2. Oligohydramnios: Less likely diagnosis given the ultrasound findings, normal pregnancy surveillance to this point, and no history of loss of amniotic fluid.
3. Intrauterine growth restriction: Less likely diagnosis given the ultrasound findings in triage and normal pregnancy surveillance to this point.

Challenge Question: None

Domains: Check all that apply

□ Professionalism

X Communication and Interpersonal Skills

□ Medical History

□ Physical Exam

□ Shared Decision-Making

X Patient Education

□ Clinical Reasoning

□ Documentation

□ Handoff

□ Presentation

□ Other:

Type and Level of Learner: Ob/Gyn resident

Case Objectives:

1. Discuss the diagnosis of IUFD with a patient, educating them on the diagnosis and answering questions.
2. Address the patient’s emotional needs during the conversation, including recognizing the patient’s high level of anxiety.
3. Educate a patient on management options for IUFD, focusing on induction of labor.

| SETTING | OB triage |
| --- | --- |
| PATIENT PROFILE | |
| Age range | 35 |
| Religious/spiritual background | All may be used |
| Sex | Female |
| Sexual orientation | Heterosexual |
| Gender expression | Woman |
| Race and ethnicity | All may be used |
| Physical description | All may be used |
| Physical limitations | All may be used |
| Patient appearance | Hospital gown |
| Moulage + location | Gravid abdomen consistent with 37 weeks gestation |
| Affect | Very anxious given how long she had to go through IVF to conceive |
| Family group | Married to her husband who is very supportive. She lives in a single family home with her husband and 2 cats. |
| Education | All may be used |
| Level of health literacy | All may be used |
| Employment | Is a teacher at the local elementary school. She has always loved working with children and can’t wait to be a mom. |
| Home | Lives in a single family home with her husband and 2 cats |
| Financial situation | All may be used |
| Insurance status | Private |
| Habits | No h/o substance use disorder. |
| Activities | All may be used |
| Typical day | All may be used |

| CASE INFORMATION | |
| --- | --- |
| Chief Concern. | Decreased fetal movement |
| Additional Concerns | None |
| THE PATIENT’S STORY | ***Do not disclose personal information unless asked directly***  The case takes place in triage. The patient is wearing a gown with an obvious gravid abdomen. An MFM ultrasound tech happens to be at bedside. She steps outside when the resident enters the room and tells the resident that she couldn’t find the FHR which prompted her to scan the patient. US reveals a confirmed IUFD.  The patient is 37 weeks pregnant. This is her first pregnancy. This was a highly desired IVF pregnancy. She and her husband went through 2 years of IVF before conceiving. They are having a baby girl and are ecstatic to finally be parents.  She has chronic hypertension and has been taking aspirin this pregnancy. She has anxiety and takes escitalopram. She has nausea and takes ondansetron occasionally. She is otherwise medically healthy. She is aware this is a high risk pregnancy given her advanced maternal age, but she has been consistently reassured by her Ob/Gyn given that everything so far this pregnancy has been uncomplicated.  All of the genetic screening through this pregnancy has been normal. She was planning on being induced in one week for her chronic hypertension. This morning, she realized she hadn’t felt her baby move as much as normal. She drank some cold apple juice and still felt no movement. She decided to come to OB triage to get checked out. She doesn’t have any abdominal pain or vaginal bleeding so she feels overall reassured. She will just feel better once she hears the baby’s heart rate on the monitor.  The resident comes to discuss the results of her ultrasound.  The resident should recognize this patient’s high anxiety.  The resident is there to discuss that her baby no longer has a heartbeat and that her baby has died.  The patient is very anxious. She states “I just want to hear my baby’s heartbeat, then I’ll feel better.”  **SP instructions:** Once the resident states that the baby no longer has a heartbeat, the patient is shocked. She is tearful and distraught, and continues to repeat something along the lines of:  “There must be a mistake”  “I was supposed to be induced next week. This can’t be right.”  “I felt her kick yesterday.”  “This can’t be right”  “Please check again.”  “Are you sure?”  “What did I do wrong?”  “Why did this happen?”  “What can you do to fix it?”  “What do we do now? What happens next?”  If asked,  Are you in any pain? No  Are you having any vaginal bleeding? No  Are you having any leakage of fluid? No  When was the last time you felt your baby move? Last night before I went to bed  Are you having any headaches? No  Are you having any vision changes? No  Are you having any right upper quadrant pain? No  Does your baby have a name? No  Do you want to discuss the options for what’s next? Sure |
| HISTORY OF PRESENT ILLNESS | |
| Onset | This morning |
| Setting | At home |
| Duration |  |
| Time relationships |  |
| Location |  |
| Radiation |  |
| Quality |  |
| Amount |  |
| Aggravated by what |  |
| Relieved by what |  |
| Associated with what |  |
| Attitude | She doesn’t have any abdominal pain or vaginal bleeding so she feels overall reassured. She will just feel better once she hears the baby’s heart rate on the monitor. |
| Overall course | Movement not increased after drinking cold apple juice |
| REVIEW OF SYSTEMS | |
| +Decreased fetal movement | -Pain |
|  | -Vaginal bleeding |
|  | -Leakage of fluid |
|  | -Headaches |
|  | -Vision changes |
|  | -RUQ pain |
| Past medical history | |
| Medication allergies (name and reaction) | None |
| Environmental allergies (name and reaction) | None |
| Illnesses | HTN, anxiety |
| Vaccinations | Up to date on vaccinations |
| Surgeries | None |
| Accidents/injuries/trauma | None |
| Hospitalization | None |
|  | |
| Inclusive sexual and reproductive history | |
| Sexual practices  Sexual partners  Protection: Use of safer sex practices  Use of birth control if appropriate  Risk of intimate partner violence | Monogamous with husband  No contraception or barrier protection  No concern for IPV |
| OB/GYN history | Age of onset of menses 13  Age of menopause NA  Number of pregnancies 4  Number of live births 0  Number of miscarriages 3  Number of abortions 0 |
| Medications | Prescription/dose/reason   - Hypertension: aspirin 81 mg - Anxiety: escitalopram - Nausea: ondansetron PRN   Over the counter/dose/reason- NA  Herbs/supplements/dose/reason- NA |
| Immunizations | X Tetanus  X Flu  X Hepatitis  □ Pneumovax  X COVID  X HPV  □ Other |
| Tobacco products  □ Cigarettes  □ Cigar  □ Pipe  □ Chew  □ E-cigarettes | X Never  □ Past - year started/year quit  □ Current  o ppd  o # of years |
| Alcohol  □ Beer  □ Wine  □ Liquor  □ Other | X Never  □ Past - year started/year quit  □ Current  o Quantity  o # of years |
| Drugs  □ Weed  □ Cocaine  □ Heroin  □ Meth  □ IV  □ Inhalants  □ Other | X Never  □ Past - year started/year quit  □ Current  o Quantity  o # of years |
| Diet | All may be used |
| Exercise | All may be used |
| List any other important social history or information important to this case | NA |
| Family history |  |
| Mother, father, siblings, grandparents, and other significant findings | Father: T2DM  Mother: HTN  MGM: h/o breast cancer |
|  |  |
| Physical Exam  No physical exam conducted | |
|  |  |
| DIAGNOSIS AND DIFFERENTIAL | |
| Diagnosis with support from positive and negative history and PE findings | Intrauterine fetal demise: Most likely diagnosis given clinical history of decreased fetal movement and lack of a heartbeat detected on Doppler and ultrasound. |
| Differential with support from positive and negative history and PE findings | Oligohydramnios: Less likely diagnosis given the ultrasound findings, normal pregnancy surveillance to this point, and no history of loss of amniotic fluid.  Intrauterine growth restriction: Less likely diagnosis given the ultrasound findings in triage and normal pregnancy surveillance to this point. |
|  |  |
| MANAGEMENT OR DIAGNOSTIC PLAN | |
|  | Discuss diagnosis of intrauterine fetal demise. Address emotional concerns first. If time allows, can discuss induction of labor but this is not a necessity. If time allows to discuss management, the patient understands the diagnosis and wishes to proceed with induction of labor. |
| PROFESSIONALISM ISSUES OR CHALLENGES | In addition to delivering the difficult news of an IUFD at term to the patient, learners must be able to address the emotional concerns of the patient. In particular, this patient conceived following two years of IVF with her husband and has a history of three prior miscarriages. The patient also has a known history of anxiety and is on medication for her symptoms. This context must be taken into account during the patient counseling. |
