# Supplementary material for: Breaking Bad News: A Simulation-Based Training Program for OB/GYN Residents
Source: MedEdPORTAL. 2026 Jun 4;22:11606. doi: 10.15766/mep_2374-8265.11606 (PMC13233813; doi:10.15766/mep_2374-8265.11606)
Supplement: Supplementary file 1 — Palliative Care Didactic.pptxCase 1 - Previable Preterm Prelabor Rupture.docxCase 2 - Surgical Complication.docxCase 3 - Cancer Diagnosis.docxCase 4 - Intrauterine Fetal Demise.docxPre- and Postsession Questionnaires.docx [file mep_2374-8265.11606-s001.zip › F. Pre- and Postsession Questionnaires.docx]

**Pre- and Postsession Questionnaire**

*This questionnaire was administered to learners both before and after the simulation session. The same questionnaire was used at both time points.*

**Demographics**

1. What is your current year of residency training?

- PGY-1
- PGY-2
- PGY-3
- PGY-4

**Open-ended Question**

1. In the last month, how many times did you break bad news? (*Free text)*

**Affirmations Using a Scale**

Please answer the questionnaire below about how you usually break bad news:

1. If I am performing an ultrasonography examination, I turn on the lights to report bad news.

- Totally disagree
- Partially disagree
- Neither agree nor disagree
- Partially agree
- Totally agree

2. I ask patients to sit down before delivering bad news.

- Totally disagree
- Partially disagree
- Neither agree nor disagree
- Partially agree
- Totally agree

3. I request that patients get dressed before delivering bad news.

- Totally disagree
- Partially disagree
- Neither agree nor disagree
- Partially agree
- Totally agree

4. I take patients to another room to report the bad news.

- Totally disagree
- Partially disagree
- Neither agree nor disagree
- Partially agree
- Totally agree

5. I consider the privacy of the environment in which the bad news is communicated to be important.

- Totally disagree
- Partially disagree
- Neither agree nor disagree
- Partially agree
- Totally agree

6. If a patient is alone, I ask if they want to be accompanied by someone before communicating bad news.

- Totally disagree
- Partially disagree
- Neither agree nor disagree
- Partially agree
- Totally agree

7. When I break bad news, I first think about what I will say.

- Totally disagree
- Partially disagree
- Neither agree nor disagree
- Partially agree
- Totally agree

8. I feel comfortable breaking bad news.

- Totally disagree
- Partially disagree
- Neither agree nor disagree
- Partially agree
- Totally agree

9. I feel calm about breaking bad news.

- Totally disagree
- Partially disagree
- Neither agree nor disagree
- Partially agree
- Totally agree

10. I feel prepared to break bad news.

- Totally disagree
- Partially disagree
- Neither agree nor disagree
- Partially agree
- Totally agree

11. I have the ability to break bad news.

- Totally disagree
- Partially disagree
- Neither agree nor disagree
- Partially agree
- Totally agree

12. Before communicating bad news, I ask the patient what they know about the situation.

- Totally disagree
- Partially disagree
- Neither agree nor disagree
- Partially agree
- Totally agree

13. I have the ability to deliver bad news about pregnancies.

- Totally disagree
- Partially disagree
- Neither agree nor disagree
- Partially agree
- Totally agree

14. I have the ability to deliver bad news about gynecology.

- Totally disagree
- Partially disagree
- Neither agree nor disagree
- Partially agree
- Totally agree

15. I have the ability to deliver bad news about gynecology oncology.

- Totally disagree
- Partially disagree
- Neither agree nor disagree
- Partially agree
- Totally agree

16. I ask how much information the patient wants before communicating the information.

- Totally disagree
- Partially disagree
- Neither agree nor disagree
- Partially agree
- Totally agree

17. I am skilled in discussing the diagnosis.

- Totally disagree
- Partially disagree
- Neither agree nor disagree
- Partially agree
- Totally agree

18. I have the ability to discuss the prognosis.

- Totally disagree
- Partially disagree
- Neither agree nor disagree
- Partially agree
- Totally agree

19. I am skilled in talking about the end of pregnancy or the beginning of palliative care.

- Totally disagree
- Partially disagree
- Neither agree nor disagree
- Partially agree
- Totally agree

20. I am skilled in discussing issues related to the end of life.

- Totally disagree
- Partially disagree
- Neither agree nor disagree
- Partially agree
- Totally agree

21. I feel confident answering difficult questions asked by patients during the communication of bad news.

- Totally disagree
- Partially disagree
- Neither agree nor disagree
- Partially agree
- Totally agree

21. I had specific training on how to report bad news.

- Totally disagree
- Partially disagree
- Neither agree nor disagree
- Partially agree
- Totally agree

22. I consider the use of a strategy or protocol to communicate bad news important.

- Totally disagree
- Partially disagree
- Neither agree nor disagree
- Partially agree
- Totally agree

23. I have knowledge about the communication of bad news.

- Totally disagree
- Partially disagree
- Neither agree nor disagree
- Partially agree
- Totally agree

If you have experience with any protocol for delivering bad news, answer the following questions:

24. Although I know a communication protocol for bad news, I prefer to use my own clinical practices to communicate bad news.

- Totally disagree
- Partially disagree
- Neither agree nor disagree
- Partially agree
- Totally agree

25. I believe that the use of this protocol helps in clinical practice.

- Totally disagree
- Partially disagree
- Neither agree nor disagree
- Partially agree
- Totally agree

26. Please briefly describe the protocol for breaking bad news that you know or use. (*Free text*)

*Adapted from the Questionnaire for the Analysis of Perceptions of Breaking Bad News Specific to the Obstetric Area (QAPBBN-O), Oliveira FF, Benute GRG, Gibelli MAB, et al. Breaking Bad News: A Study on Formal Training in a High-Risk Obstetrics Setting. Palliative Medicine Reports. 2020;1(1):50-57. doi:10.1089/pmr.2020.0014*
