# Supplementary material for: Breaking Bad News: A Simulation-Based Training Program for OB/GYN Residents
Source: MedEdPORTAL. 2026 Jun 4;22:11606. doi: 10.15766/mep_2374-8265.11606 (PMC13233813; doi:10.15766/mep_2374-8265.11606)
Supplement: Supplementary file 1 — Palliative Care Didactic.pptxCase 1 - Previable Preterm Prelabor Rupture.docxCase 2 - Surgical Complication.docxCase 3 - Cancer Diagnosis.docxCase 4 - Intrauterine Fetal Demise.docxPre- and Postsession Questionnaires.docx [file mep_2374-8265.11606-s001.zip › _Educational Summary Report_11606.pdf]

MedEdPORTAL
THE AAMC JOURNAL OF TEACHING AND LEARNING RESOURCES

Original Publication

Open Access

Breaking Bad News: A Simulation-Based Training Program for OB/GYN Residents

Marisa R. Imbroane*, Katarina Fleckenstein, MD, Haley Vest, DO, Erin Higgins, MD

*Corresponding author: mri13@case.edu

Abstract

Introduction: Delivering bad news has ethical, social, and medicolegal obligations for medical professionals. The way bad news is delivered directly affects patients' understanding and satisfaction with their medical care, with poor communication skills and emotional strain contributing to patient dissatisfaction and provider stress. Obstetrics and gynecology residents are often responsible for breaking bad news to patients, but there are few formal training programs focused on developing this skill. **Methods:** We developed a simulation-based curriculum in delivering bad news for obstetrics and gynecology residents. The 3-hour curriculum consisted of a short didactic on palliative medicine, case-based standardized patient interaction, and a panel discussion with patients and health care professionals. Case topics included previable preterm prelabor rupture of membrane, surgical complication, cancer diagnosis, and intrauterine fetal demise. Participants completed a questionnaire based on the SPIKES protocol before and after training. Responses were compared pre- and posttraining to evaluate for change in participants' perceptions of their own ability to deliver bad news. **Results:** Fifteen obstetrics and gynecology residents, from PGY 1 to PGY 4, completed the pretraining questionnaire, and 13 completed the posttraining questionnaire. Posttraining surveys indicated that residents felt more prepared to deliver bad news compared to prior to training. **Discussion:** This pilot study demonstrates that a simulation-based curriculum on breaking bad news can improve residents' ability to deliver bad news. Additionally, this course could be adapted for other specialties, such as general surgery and internal medicine, to address this educational need.

Keywords
Communication, Bad News, Simulation, Standardized Patient, SPIKES, OB/GYN

Educational Objectives

By the end of this activity, learners will be able to:

1. Describe and demonstrate their professional responsibility when delivering bad news to patients.
2. Apply the SPIKES protocol to structure a bad news conversation, including setting up the interview, assessing patient perception, obtaining an invitation to discuss the news, delivering knowledge, addressing patient emotions, and summarizing next steps.
3. Demonstrate effective responses to patient emotions following the delivery of bad news, using empathetic statements, reflective listening, and supportive silence.

Citation:
Imbroane MR, Fleckenstein K, Vest H, Higgins E. Breaking bad news: a simulation-based training program for OB/GYN residents. *MedEdPORTAL*. 2026;22:11606.
https://doi.org/10.15766/mep_2374-8265.11606

Introduction

Delivering bad news to patients is an essential skill in a physician's repertoire that has moral, social, and ethical implications. The way difficult news is delivered can directly impact a patient's understanding and satisfaction of their medical care.[1] Additionally, poor communication skills when delivering bad news have been shown to contribute to both patient dissatisfaction and provider stress.[2] Despite the importance of this skill, many medical trainees and physicians report limited formal training in breaking bad news and feel inadequately prepared for these conversations.[3,4] Studies also demonstrate variability, and even deficiencies, among physician communication skill performance.[5,6] Therefore, this is a clinical skill that needs to be included in the medical curriculum, especially at the resident level when new clinicians begin to deliver difficult news.

Residents in the field of obstetrics and gynecology (OB/GYN) regularly face the challenge of delivering bad news. Whether it

Copyright © 2026 Imbroane et al. This is an open-access publication distributed under the terms of the Creative Commons Attribution license.

be counseling patients on abnormal anatomy scans, diagnosing gynecologic malignancies, discussing surgical complications, or informing a patient that their pregnancy is no longer viable, obstetricians and gynecologists must share difficult news daily with patients and their families. Yet, curricula to prepare OB/GYN residents for this challenge is scant in the literature, despite an understood importance on developing general communication skills for medical student and resident learners.[5,7,8] There are several studies on simulation-based curricula for breaking bad news within OB/GYN training, including studies on curricula for delivering news of perinatal loss and the efficacy of debrief versus lecture following simulation session.[9,10] However, these studies lack curriculum materials that institutions could download and adopt for their learners.[9,10] A large volume of the education in breaking bad news is grounded in the SPIKES protocol, which stands for Setting, Perception, Invitation, Knowledge, Emotion, and Strategy and Summary.[3] Research has suggested that the SPIKES protocol could be useful within the field of OB/GYN and that residents find it useful for learning specific communication skills.[11,12] However, more evidence is needed to show whether SPIKES can improve resident performance in communicating difficult news to patients.

MedEdPORTAL has published several teaching resources which utilize the SPIKES protocol for learners at various stages of their medical training. For medical students, there are curricula available covering preclerkship communication skills, including a specific standardized patient (SP) session on delivering a miscarriage diagnosis.[13,14] Several curricula are also available for the resident or fellow level (including for oncology and neonatal-perinatal fellows), but none focus on the OB/GYN scope of practice specifically.[15,16] Therefore, there is a need for both the development of curriculum aimed specifically at helping OB/GYN residents improve their technique in delivering bad news and to assess whether resident learners find the SPIKES protocol to be a useful tool for this communication skill.

To address this curricular gap, we designed a breaking bad news curriculum for OB/GYN residents at our institution that covered 4 different SP scenarios: previable preterm prelabor rupture of membranes (PPROM), (2) surgical complication, (3) cancer diagnosis, and (4) intrauterine fetal demise (IUFD). The curriculum was a combination of a palliative medicine didactic with training on the SPIKES protocol, an SP simulation experience, and a panel discussion. We assessed the self-perceived ability of OB/GYN residents to deliver difficult news to patients both before and after completing the 3-hour curriculum.

Methods

Activity Design and Development

The curriculum was designed by a resident with assistance from an OB/GYN faculty member with formal simulation training. The cases were selected from real clinical scenarios encountered by residents in our program and then refined with assistance from SP educators.

The curriculum design was guided by Kolb's Experiential Learning Theory, which provided the basis for the course as it progressed from didactic (abstract conceptualization) to an SP interaction with formal debriefing (active experimentation with reflective debriefing) and ending with a panel discussion (concrete experience).[17] Additionally, principles of deliberate practice were incorporated into the SP encounter, where the interaction was observed and learners received immediate, directed feedback from faculty.[18]

Curricular Context and Learner Preparation

All learner participants were OB/GYN residents and represented all 4 years of residency training. This course took place during protected resident education time, as part of the 2-year simulation curriculum. Facilitators did not require additional preparation.

This study was submitted to and approved by the Cleveland Clinic Institutional Review Board: Simulation-Based Training in OB/GYN (23-405).

Equipment

The following equipment was necessary for the implementation of the curriculum:

- Objective Structured Clinical Examination (OSCE) rooms (4)
- Chairs for each learner group
- Gown for cases

Personnel

Four facilitators participated, 1 for the didactic, who also covered a simulation case, and 3 additional facilitators for the simulation. Four SPs were used. The panel discussion consisted of community members who were recruited through local advocacy organizations (eg, cancer survivors' groups) and personal connections. Panelists introduced themselves and shared a brief account of their experience receiving bad news from a medical professional. Panelists in our session had experienced pregnancy loss and cancer diagnoses. Learners were given the opportunity to ask questions of the panelists to better understand their experiences and the impact of physician communication.

Copyright © 2026 Imbroane et al. This is an open-access publication distributed under the terms of the Creative Commons Attribution license.

Implementation

The total duration of the curriculum was 3 hours and began with a prebrief conducted by the lead simulation faculty for this course. The prebrief included a review of The Basic Assumption, flow of day, psychological safety of learners and SPs, physical safety of SPs, and logistical aspects such as bathroom locations.[19] Following the prebrief, learners observed a 15-minute didactic on palliative medicine and delivering bad news given by a palliative care physician, which focused on the SPIKES protocol (Appendix A). This lecture, along with the simulation prebrief, was completed in a classroom at the Health Education Campus of Case Western Reserve University (CWRU).

The learners then moved to the CWRU Clinical Skills and Simulation Center, where they participated in 4 case-based encounters with SPs (Appendices B-E). These encounters were held in OSCE rooms equipped with audiovisual equipment and 2-way mirrors. The topics for the SP encounters were (1) previable PPROM, (2) surgical complication, (3) cancer diagnosis, and (4) IUFD. These topics were chosen by simulation faculty in collaboration with resident input to select relevant cases. SPs were recruited and trained by simulation education specialists from the Cleveland Clinic Simulation and Advanced Skills Center. Each case had 1 faculty facilitator recruited from our organization based on their areas of expertise and experience in delivering bad news. Our faculty members consisted of 2 gynecologic oncologists, 1 maternal-fetal medicine specialist, and 1 palliative care hospitalist. Faculty were provided with the PEARLS Healthcare Debriefing Tool to aid their debriefing sessions.[20] Our organization also offers online and in-person facilitator training for those interested, although this was not a requirement of faculty participating in this course.

Learners were divided into small groups of 4 to 6 learners, with 1 resident participating in each SP encounter. The other learners and faculty observed each encounter from outside the room. A brief overview of the presenting chief complaint was described on a door note prior to entry into the room. At the end of the encounter, the SP was given 2 minutes to provide verbal feedback on the interaction, with the option to provide additional written feedback after exiting the OSCE space. A formal debrief with faculty and other group members then took place in the OSCE room. During the debrief, the observing learners were reminded of The Basic Assumption and the goal of “want[ing] to improve.”[19] Learners were encouraged to focus their discussion on the facts of the case and share their experiences in similar situations rather than criticize the performance of their peers. Each session was 30 minutes in duration, for a total of 2 hours for the entire simulation experience.

After rotating through the simulation encounters, learners returned to the classroom for a 1-hour panel discussion with community members. The panel was moderated by faculty and consisted of each panelist sharing a brief personal story related to receiving bad news in a health care setting. Learners were able to ask questions of the panelists to explore more about the positive and negative aspects of their experiences.

Evaluation

Before and after the curricular session, the learners completed questionnaires based on the SPIKES protocol that inquired about their self-perceived ability to deliver difficult news. The questionnaire was adapted from the questionnaire for the analysis of perceptions of breaking bad news specific to the obstetric area (QAPBBN-O) of Oliveira et al.[21] The QAPBBN-O was developed within the OB/GYN department at the University of Sao Paulo based on a literature review of the main perceptions of health care professionals on breaking bad news and was designed to model the SPIKES protocol. Then, the questionnaire was finalized by a multidisciplinary team composed of providers from obstetrics, palliative medicine, nursing, social work, and psychology. For our curricula, we used all scale questions from the QAPBBN-O but added 2 questions to better reflect this simulation experience: comfort with delivering bad news for gynecology and gynecology oncology. We did not use the demographic questions from the QAPBBN-O and only adopted 1 of the 2 open-ended questions to reduce time needed to complete the questionnaire. In total, our learners were asked their current year of residency training, how many times they delivered bad news in the past month (open-ended), the level to which they agreed or disagreed with 25 statements on a 5-point scale, and to describe a protocol they knew or used for breaking bad news (Appendix F). We compared the level of perceived self-confidence before and after the curricular experience. Due to the capability of inferential statistics being limited by our sample size, only descriptive statistics were performed. On the postsession questionnaire, participants were also given the option to leave open-ended feedback regarding their experience with the simulation session. Open-ended comments regarding learner experience with the simulation session were assessed by the curriculum design team. These comments were used to gauge areas of improvement in future iterations of the simulation session.

Copyright © 2026 Imbroane et al. This is an open-access publication distributed under the terms of the Creative Commons Attribution license.

Results

Overall, 15 residents participated in the curricular session, and 13 completed both the pre- and postsession evaluations (a response rate of 86%). Of the 13, 5 (39%) were PGY 1, 4 (31%) were PGY 2, 3 (23%) were PGY 3, and 1 (8%) was PGY 4. Results of the pre- and postsession Likert-type items from the questionnaires are shown in Table 1.

Prior to the curriculum, no learners strongly agreed that they had received specific training on how to deliver bad news to patients. This increased to 100% of participants strongly agreeing after the session. Additionally, only 8% of learners totally agreed and 69% partially agreed that they had knowledge about the communication of bad news before the session. This increased to 77% totally agreeing and the remaining 23% now partially

Table 1. Pre- and Postsession Survey Results ($N = 13$)

	Response No. (%)											
	Not Applicable		Totally Disagree		Partially Disagree		Neither Agree nor Disagree		Partially Agree		Totally Agree	
**Item**	**Pre**	**Post**	**Pre**	**Post**	**Pre**	**Post**	**Pre**	**Post**	**Pre**	**Post**	**Pre**	**Post**
**If I am performing an ultrasonography examination, I turn on the lights to report bad news.**	1 (8)	0 (0)					3 (23)	1 (8)	2 (15)	2 (15)	7 (54)	10 (77)
**I ask patients to sit down before delivering bad news.**	1 (8)	0 (0)							2 (15)	1 (8)	10 (77)	12 (92)
**I request that the pregnant patient get dressed before delivering bad news.**					2 (15)	0 (0)	2 (15)	1 (8)	3 (23)	4 (31)	6 (46)	8 (62)
**I take pregnant women to another room to report the bad news.**					4 (31)	2 (15)	7 (54)	3 (23)	2 (15)	7 (54)	0 (0)	1 (8)
**I consider the privacy of the environment in which the bad news is communicated to be important.**											13 (100)	13 (100)
**If a patient is alone, I ask if she wants to be accompanied by someone before communicating bad news.**					1 (8)	0 (0)			8 (62)	1 (8)	4 (31)	12 (92)
**When I break bad news, I first think about what I will say.**									2 (15)	0 (0)	11 (85)	13 (100)
**I feel comfortable breaking bad news.**					5 (39)	1 (8)	1 (8)	2 (15)	7 (54)	9 (69)	0 (0)	1 (8)
**I feel calm about breaking bad news.**					4 (31)	1 (8)	2 (15)	3 (23)	6 (46)	6 (46)	1 (8)	3 (23)
**I feel prepared to break bad news.**					4 (31)	0 (0)	3 (23)	1 (8)	5 (39)	7 (54)	1 (8)	5 (39)
**I have the ability to break bad news.**							1 (8)	0 (0)	9 (70)	6 (46)	3 (23)	7 (54)
**Before communicating bad news, I ask the patient what she knows about the situation.**									3 (23)	4 (31)	10 (77)	9 (70)
**I have the ability to deliver bad news about pregnancies.**					2 (15)	2 (15)	3 (23)	0 (0)	5 (39)	3 (23)	3 (23)	8 (62)
**I have the ability to deliver bad news about gynecology.**					0 (0)	2 (15)	3 (23)	0 (0)	10 (77)	4 (31)	0 (0)	7 (54)
**I have the ability to deliver bad news about gynecology oncology.**					5 (39)	2 (15)	1 (8)	0 (0)	5 (39)	6 (46)	2 (15)	5 (39)
**I must know how much information the patient wants before communicating the information.**							0 (0)	1 (8)	6 (46)	3 (23)	7 (54)	9 (70)
**I am skilled in discussing the diagnosis.**			1 (8)	1 (8)	4 (31)	1 (8)	2 (15)	1 (8)	5 (39)	7 (54)	1 (8)	3 (23)
**I have the ability to discuss the prognosis.**			2 (15)	1 (8)	4 (31)	3 (23)	2 (15)	1 (8)	5 (39)	6 (46)	0 (0)	2 (15)
**I am skilled in talking about the end of pregnancy or the beginning of palliative care.**			2 (15)	1 (8)	3 (23)	3 (23)	2 (15)	2 (15)	6 (46)	5 (39)	0 (0)	2 (15)
**I am skilled in discussing issues related to the end of life.**			1 (8)	1 (8)	2 (15)	2 (15)	5 (39)	1 (8)	5 (39)	7 (54)	0 (0)	2 (15)
**I feel confident answering difficult questions asked by patients during the communication of bad news.**			2 (15)	1 (8)	4 (31)	1 (8)	1 (8)	0 (0)	6 (46)	8 (62)	0 (0)	3 (23)
**I had specific training on how to report bad news.**			2 (15)	0 (0)	2 (15)	0 (0)			9 (70)	3 (23)	0 (0)	10 (77)
**I consider the use of a strategy or protocol to communicate bad news important.**					1 (8)	1 (8)	2 (15)	1 (8)	7 (54)	3 (23)	3 (23)	8 (62)
**I have knowledge about the communication of bad news**					1 (8)	0 (0)	2 (15)	0 (0)	9 (70)	3 (23)	1 (8)	10 (77)

Copyright © 2026 Imbroane et al. This is an open-access publication distributed under the terms of the Creative Commons Attribution license.

agreeing after the session. Comparing pre- and postsession survey results, residents felt more prepared when breaking bad news (46% vs 85% saying that they partially agreed or totally agreed). Specifically, residents felt more comfortable delivering bad news related to IUFD (61% vs 84%), cancer diagnosis (38% vs 69%), and previable PPROM (46% vs 77%), all reported as participants responding that they partially agreed or totally agreed. One hundred percent of participants stated that they felt this training helped in their clinical practice. At the end of the session, 77% of participants disagreed with the statement "Although I know a communication protocol for bad news, I prefer to use my own clinical practices to communicate bad news," and 100% of participants agreed that the SPIKES protocol helps in clinical practice (Table 2).

Common ideas identified in the feedback included real-life applicability and appreciation of a safe space to practice these skills. Selected responses are presented next:

- "The sim was amazing! It was a grounding experience and refocused the importance and impact of what we do on a daily basis. Thank you for this training that I will take with me throughout my career."
- "This was a really great hands-on experience that gave me skills I can apply in real life."
- "These topics are HARD. Thank you for allowing us a safe space to practice. I now have a mental checklist I can go through before I walk into a room to have that tough conversation."
- "I wish I had had this earlier in my residency career!"

Discussion

The ability to deliver bad news is an essential skill for OB/GYN physicians, yet specific training during residency is seldom described in existing literature.[7] Therefore, we developed a curriculum to address this need, filling the gap of simulation experiences for learners by creating 4 SP scenarios that range topics in general OB/GYN, gynecology, and high-risk antepartum care. The structure of this course allowed learners to progress through Kolb's Experiential Learning Cycle, abstract conceptualization during the didactic session, followed by active experimentation with reflective debriefing in the SP encounter. Finally, the panel discussion provided a concrete experience in which learners heard from the real-life experiences of patients, which is likely something they will encounter in the clinical realm as well. Our evaluation of the curricula demonstrates that our session was effective in improving residents' self-perceived ability to deliver bad news. Thus, we plan to offer this training on a regular basis as part of our educational programming and hope to see it adapted by other OB/GYN residency programs. Additionally, we believe that this curriculum could be adapted for other specialties, as literature regarding resident education on delivering bad news is limited across the medical field.

Previous literature has indicated positive feedback on the use of the SPIKES protocol among OB/GYN residents.[3] Our curriculum was also very well received by residents, with all participants believing that the SPIKES protocol is useful in clinical practice. However, we were unable to assess whether resident communication skills were actually improved or if the self-perception in improvement was inflated directly following the training. Setubal et al.[9] have found that education on the SPIKES protocol itself did not improve SP feedback of residents' ability to deliver bad news. Although it would be ideal to measure patient comfort while receiving difficult news, it is known that physicians face stress as well when needing to deliver difficult news.[11] Therefore, being able to improve residents' comfort with delivering bad news and seeing that they are more likely to feel calm when delivering difficult news is an important outcome of the training. However, in future iterations of the session, we would like to incorporate SP feedback, as this feedback from patients on advanced communication skills is of high value to learners. Additionally, while most participants were highly satisfied with the curricular design, we did receive feedback that the observed patient encounters could be distressing to some learners. To mitigate this, we plan to provide a checklist to faculty, SPs, and observing participants to better guide their evaluation of the encounter and subsequent feedback. Formal SP evaluations,

Table 2. Pre- and Postsession Survey Results ($N = 13$)

	Response No. (%)					
	Not Applicable		False		True	
Item	Pre	Post	Pre	Post	Pre	Post
**Although I know a communication protocol for bad news, I prefer to use my own clinical practices to communicate bad news.**						
	8 (62)	0 (0)	5 (39)	10 (77)	0 (0)	3 (23)
**I believe that the use of this protocol helps in clinical practice.**						
	8 (62)	0 (0)	0 (0)	0 (0)	5 (39)	13 (100)

Copyright © 2026 Imbroane et al. This is an open-access publication distributed under the terms of the Creative Commons Attribution license.

provided to the participating resident in that case, would be valuable for that learner's personal development. Additionally, we will provide guidance to participants on giving peer feedback prior to the session.

The main limitation of our study was our sample size. Additionally, this cohort was overrepresented by PGY-1 and PGY-2 residents (10 out of 15 participants), which may have impacted our results. As we continue to implement this curriculum, we plan to gather ongoing feedback to adjust the session as necessary. However, given that survey responses were overall improved after completing the session, we are confident that the time spent in this session was beneficial for the learners. The free-text responses collected also indicated that this is a session we should continue to offer in the educational curriculum. Finally, as we have not yet done a follow-up survey, we are unsure if improved residents' confidence and self-reported communication skills are maintained over time. Therefore, we understand that the data would be strengthened with a time-delayed survey of resident comfort with delivering bad news and will consider administering a 6-month follow-up survey during future iterations of this simulation session.

Ultimately, we developed a 3-part curriculum for OB/GYN residents on delivering bad news. The combination of a didactic on the SPIKES protocol, 4 simulation-based patient encounters, and a panel discussion was effective at improving residents' self-perceived ability to deliver difficult news to their patients. Given our results, this curriculum would be a useful asset to include in OB/GYN residency education throughout the country.

Appendices

A. Palliative Care Didactic.pptx

B. Case 1 - Previable Preterm Prelabor Rupture.docx

C. Case 2 - Surgical Complication.docx

D. Case 3 - Cancer Diagnosis.docx

E. Case 4 - Intrauterine Fetal Demise.docx

F. Pre- and Postsession Questionnaires.docx

All appendices are peer reviewed as integral parts of the Original Publication.

Marisa R. Imbroane: Medical Student, Case Western Reserve University School of Medicine; https://orcid.org/0009-0008-8259-635X

Katarina Fleckenstein, MD: Attending Physician, Department of Obstetrics and Gynecology, St. Joseph's/Candler Physician Network

Haley Vest, DO: Assistant Professor, Department of Hospice and Palliative Care, University of Virginia School of Medicine; https://orcid.org/0009-0006-7502-2978

Erin Higgins, MD: Clinical Assistant Professor, Obstetrics and Gynecology Institute, Cleveland Clinic; https://orcid.org/0009-0001-7312-2688

Disclosures
None to report.

Funding/Support
None to report.

Ethical Approval
The Cleveland Clinic Institutional Review Board reviewed this project.

References

1. Konstantis A, Exiara T. Breaking bad news in cancer patients. *Indian J Palliat Care*. 2015;21(1):35-38. https://doi.org/10.4103/0973-1075.150172
2. Brown RF, Bylund CL. Communication skills training: describing a new conceptual model. *Acad Med*. 2008;83(1):37-44. https://doi.org/10.1097/ACM.0b013e31815c631e
3. Baile WF, Buckman R, Lenzi R, Glober G, Beale EA, Kudelka AP. SPIKES—a six-step protocol for delivering bad news: application to the patient with cancer. *Oncologist*. 2000;5(4):302-311. https://doi.org/10.1634/theoncologist.5-4-302
4. Ptacek JT, Eberhardt TL. Breaking bad news. a review of the literature. *JAMA*. 1996;276(6):496-502.
5. Rosenbaum ME, Ferguson KJ, Lobas JG. Teaching medical students and residents skills for delivering bad news: a review of strategies. *Acad Med*. 2004;79(2):107-117. https://doi.org/10.1097/00001888-200402000-00002
6. Fallowfield L, Jenkins V, Farewell V, Saul J, Duffy A, Eves R. Efficacy of a Cancer Research UK communication skills training model for oncologists: a randomised controlled trial. *Lancet*. 2002;359(9307):650-656. https://doi.org/10.1016/S0140-6736(02)07810-8
7. Ribeiro LSC, D'Abreu BF, Santiago AE, et al. Breaking bad news in obstetrics and gynecology: we must talk about it. *Rev Bras Ginecol Obstet*. 2022;44(6):621-628. https://doi.org/10.1055/s-0042-1742316
8. Kapadia MR, Lee E, Healy H, Dort JM, Rosenbaum ME, Newcomb AB. Training surgical residents to communicate with their patients: a scoping review of the literature. *J Surg Educ*. 2021;78(2):440-449. https://doi.org/10.1016/j.jsurg.2020.08.018
9. Setubal MSV, Antonio MÂRGM, Amaral EM, Boulet J. Improving perinatology residents' skills in breaking bad news: a randomized

Copyright © 2026 Imbroane et al. This is an open-access publication distributed under the terms of the Creative Commons Attribution license.

intervention study. *Rev Bras Ginecol Obstet*. 2018;40(3):137-146. https://doi.org/10.1055/s-0037-1621741

10. Karkowsky CE, Landsberger EJ, Bernstein PS, et al. Breaking bad news in obstetrics: a randomized trial of simulation followed by debriefing or lecture. *J Matern Fetal Neonatal Med*. 2016;29(22): 3717-3723. https://doi.org/10.3109/14767058.2016.1141888
11. Setubal MSV, Gonçalves AV, Rocha SR, Amaral EM. Breaking bad news training program based on video reviews and SPIKES strategy: what do perinatology residents think about it? *Rev Bras Ginecol Obstet*. 2017;39(10):552-559. https://doi.org/10.1055/s-0037-1604490
12. Leone D, Menichetti J, Barusi L, et al. Breaking bad news in assisted reproductive technology: a proposal for guidelines. *Reprod Health*. 2017;14(1):87. https://doi.org/10.1186/s12978-017-0350-1
13. Cvengros JA, Behel JM, Finley E, Kravitz R, Grichanik M, Dedhia R. Breaking bad news: a small-group learning module and simulated patient case for preclerkship students. *MedEdPORTAL*. 2016;12:10505. https://doi.org/10.15766/mep_2374-8265.10505
14. Prasad L, Hockstein S, Safdieh JE, Harvey K, Christos PJ, Kang Y. An objective structured clinical exam on breaking bad news for clerkship students: in-person versus remote standardized patient approach. *MedEdPORTAL*. 2023;19:11323. https://doi.org/10.15766/mep_2374-8265.11323
15. Janice-Woods Reed D, Sharma J. Delivering difficult news and improving family communication: simulation for neonatal-perinatal fellows. *MedEdPORTAL*. 2016;12:10467. https://doi.org/10.15766/mep_2374-8265.10467
16. Cannone D, Atlas M, Fornari A, Barilla-LaBarca ML, Hoffman M. Delivering challenging news: an illness-trajectory communication curriculum for multispecialty oncology residents and fellows. *MedEdPORTAL*. 2019;15:10819. https://doi.org/10.15766/mep_2374-8265.10819
17. Kolb D. *Experiential Learning: Experience as the Source of Learning and Development*. Prentice Hall; 1984.
18. Wang JM, Zorek JA. Deliberate practice as a theoretical framework for interprofessional experiential education. *Front Pharmacol*. 2016;7:188. https://doi.org/10.3389/fphar.2016.00188
19. The Basic Assumption. Center for Medical Simulation. Accessed March 16, 2026. https://harvardmedsim.org/resources/the-basic-assumption
20. Bajaj K, Meguerdichian M, Thoma B, Huang S, Eppich W, Cheng A. The PEARLS Healthcare Debriefing Tool. *Acad Med*. 2018; 93(2):336. https://doi.org/10.1097/ACM.0000000000002035
21. Oliveira FF, Benute GRG, Gibelli MAB, et al. Breaking bad news: a study on formal training in a high-risk obstetrics setting. *Palliat Med Rep*. 2020;1(1):50-57. https://doi.org/10.1089/pmr.2020.0014

Received: October 29, 2025
Accepted: April 16, 2026
Published: June 4, 2026

Copyright © 2026 Imbroane et al. This is an open-access publication distributed under the terms of the Creative Commons Attribution license.
